# Natural Antioxidant By-Product Mixture Counteracts the Effects of Aflatoxin B1 and Ochratoxin A Exposure of Piglets after Weaning: A Proteomic Survey on Liver Microsomal Fraction

**DOI:** 10.3390/toxins15040299

**Published:** 2023-04-19

**Authors:** Roua Gabriela Popescu, George Cătălin Marinescu, Andreea Luminița Rădulescu, Daniela Eliza Marin, Ionelia Țăranu, Anca Dinischiotu

**Affiliations:** 1 Department of Biochemistry and Molecular Biology, Faculty of Biology, University of Bucharest, Splaiul Independentei No. 91-95, 050095 Bucharest, Romania; 2 Independent Research Association, Timisului No. 58, 012416 Bucharest, Romania; 3 Blue Screen SRL, Timisului No. 58, 012416 Bucharest, Romania; 4 Laboratory of Animal Biology, National Institute for Research and Development for Biology and Animal Nutrition, Calea Bucuresti No. 1, 077015 Balotesti, Romania

**Keywords:** piglets, mycotoxins, proteomics, SWATH, DIA, protein-protein interaction network, pathway analysis, feed additives, antioxidant effect, antioxidants proteomics

## Abstract

Mycotoxins are toxic compounds produced by certain strains of fungi that can contaminate raw feed materials. Once ingested, even in small doses, they cause multiple health issues for animals and, downstream, for people consuming meat. It was proposed that inclusion of antioxidant-rich plant-derived feed might diminish the harmful effects of mycotoxins, maintaining the farm animals’ health and meat quality for human consumption. This work investigates the large scale proteomic effects on piglets’ liver of aflatoxin B1 and ochratoxin A mycotoxins and the potential compensatory effects of grapeseed and sea buckthorn meal administration as dietary byproduct antioxidants against mycotoxins’ damage. Forty cross-bred TOPIGS-40 hybrid piglets after weaning were assigned to three (*n* = 10) experimental groups (A, M, AM) and one control group (C) and fed with experimental diets for 30 days. After 4 weeks, liver samples were collected, and the microsomal fraction was isolated. Unbiased label-free, library-free, data-independent acquisition (DIA) mass spectrometry SWATH methods were able to relatively quantify 1878 proteins from piglets’ liver microsomes, confirming previously reported effects on metabolism of xenobiotics by cytochrome P450, TCA cycle, glutathione synthesis and use, and oxidative phosphorylation. Pathways enrichment revealed that fatty acid metabolism, steroid biosynthesis, regulation of actin cytoskeleton, regulation of gene expression by spliceosomes, membrane trafficking, peroxisome, thermogenesis, retinol, pyruvate, and amino acids metabolism pathways are also affected by the mycotoxins. Antioxidants restored expression level of proteins PRDX3, AGL, PYGL, fatty acids biosynthesis, endoplasmic reticulum, peroxisome, amino acid synthesis pathways, and, partially, OXPHOS mitochondrial subunits. However, excess of antioxidants might cause significant changes in CYP2C301, PPP4R4, COL18A1, UBASH3A, and other proteins expression levels. Future analysis of proteomics data corelated to animals growing performance and meat quality studies are necessary.

## 1. Introduction

Mycotoxins are toxic secondary metabolites produced by certain fungi that grow on food and feed crops [1,2,3,4]. Two of the most prevalent and well-known mycotoxins are ochratoxin A (OTA) and aflatoxin B1 (AFB1) [5,6,7,8,9,10]. These toxins can contaminate food and feed products, which in turn can lead to a range of adverse effects on animal health and nutrition [11,12,13]. In pigs, the exposure to OTA and AFB1 has been shown to impair feed intake, growth performance, and immunity, as well as increase oxidative stress and the risk of various diseases [14,15,16,17].

Mycotoxins present in feed can have significant impacts on human health [18,19]. Consumption of contaminated animal products, such as meat, by human beings facilitates their exposure to mycotoxins and their secondary metabolites, causing a range of health problems, depending on the type and level of mycotoxin involved [1,20,21,22,23].

In humans, AFB1 exposure has been linked to liver cancer, as well as other health problems such as immune suppression, stunted growth, and decreased cognitive function [24,25,26,27,28,29,30]. Also, OTA has been shown to cause kidney toxicity in humans, increased risk of cancer, decreased immunity, and oxidative stress [31,32,33,34,35]. In addition to these direct health effects, mycotoxins can also impact human health by reducing the nutritional quality of animal products. For example, mycotoxins can cause decreased feed intake and reduced weight gain in animals, which can result in lower levels of key nutrients in animal-derived food products [11,36,37,38,39].

To mitigate the negative impact of mycotoxins on pig nutrition, various strategies have been proposed, including the use of antioxidants [40,41]. One promising approach is inclusion in feed of by-products like, for example, sea buckthorn and grapeseed, which are rich in antioxidants [42,43,44,45,46].

That is why the present study investigated the in-depth effects of OTA and AFB1 on the liver proteome microsomal fraction in pigs exposed to the mycotoxin mixture, as well as the capacity of a diet including a mixture of grapeseed and sea buckthorn meal to mitigate the toxicity of OTA and AFB1 co-contamination. Results of this study provide important data useful for the development of effective strategies for reducing the impact of mycotoxins on animal health and nutrition.

Proteomics studies are usually done using mass spectrometers, and ions and their collision-induced fragments are scanned as they elute from the column during a LC separation. Then, they are identified by software and mapped to peptides and proteins. Traditionally, top-most abundant ions are selected for fragmentation, this technology being named Data Dependent Acquisition (DDA) or information-dependent acquisition (IDA). However, this methodology is biased, favoring the most abundant proteins and masking the less abundant, which are also the most interesting for their regulatory functions. Recent advances in the speed and accuracy of mass spectrometers, but also in acquisition and data processing software, permit unbiased, deeper proteome coverage, using data-independent acquisition (DIA) methods. Moreover, neural networks implemented in artificial intelligence (AI) software, such as DIA-NN, permit unprecedented deconvolution, having excellent peptide mapping performance.

The present investigation employed unbiased DIA SWATH proteomics to perform large scale differential protein expression. Pathway enrichment and heatmap clustering analysis have been performed. Changes and protein interactions were also represented on KEGG pathway views in a comprehensive fashion. Our findings suggest that this approach could be an invaluable resource for forthcoming inquiries that aim to enhance our understanding of the molecular mechanisms underlying the activities of mycotoxins and antioxidants in different organisms.

In an earlier study, we found that piglets exposed to both Ochratoxin A (OTA) and Aflatoxin B1 (AFB1) had their gene expression affected at the transcriptional level. Specifically, the protein expressions of CYP2E1 and CYP3A29 were slightly up-regulated in the liver [45], whereas the enzymatic activities of CYP1A2, CYP2E1, and CYP3A29 were significantly decreased. However, when the piglets were fed with a mixture of grape seed and sea buckthorn meal, the negative effects were reduced and liver function returned to control levels [46].

In this study, we aim to investigate the effects of OTA and AFB1 on the liver at the proteomic level in pigs, showing how the addition of grape seed and sea buckthorn meal to their diet affects liver function.

## 2. Results

SWATH MS files were analyzed in library-free mode: the spectra library was generated from actual DIA data using the fasta file for the *Sus scrofa* proteome. The library contains 81,574 peptides (<1% FDR, resulting in the identification of 1878 proteins (Table S1). The median gene group coefficient of variation (at 1% FDR) was between 0.05–0.06. The identified gene groups (at 1% FDR) were between 1490–1706. The full list of identified proteins is provided in the Supplementary Material.

### 2.1. Pathway Enrichment Analysis of the Differentially Expressed Proteins

**Figure 1:**
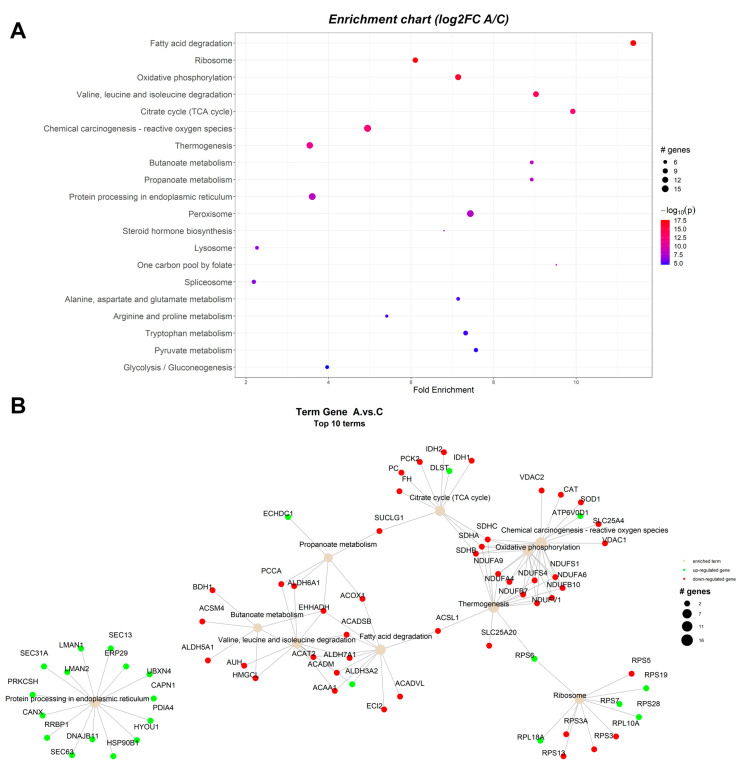
Enrichment chart (**A**) and term gene graph (**B**) from log2FC (A/C) data for the microsomal fraction from the liver of piglets. The control group (C) were fed with the basal diet. The antioxidants experimental group were fed with the basal diet plus a mixture (1:1) of two byproducts (grapeseed and sea buckthorn meal) (A group). Top 20 KEGG Pathways sorted by lowest *p* value are shown.

**Figure 2:**
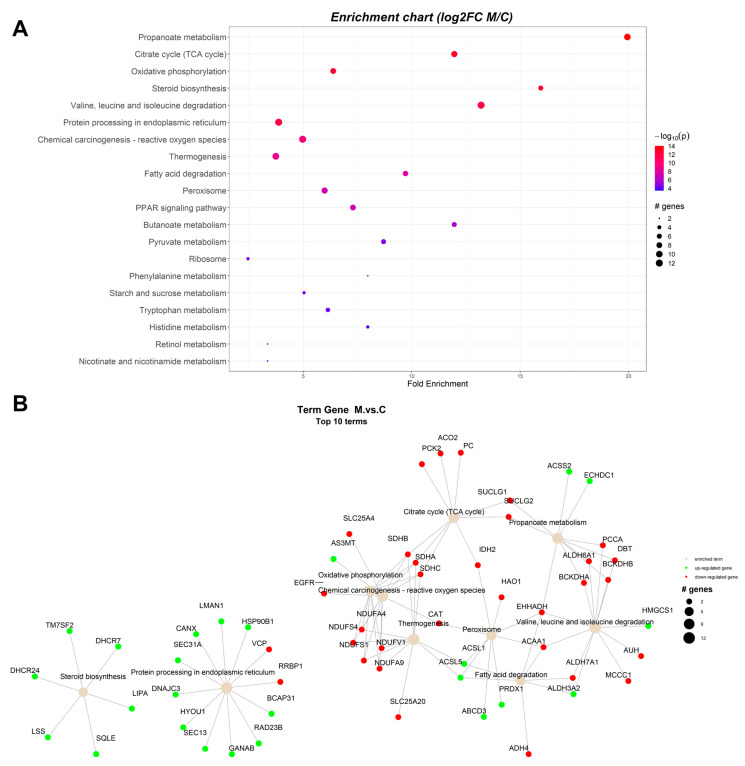
Enrichment chart (**A**) and term gene graph (**B**) from log2FC (M/C) data for the microsomal fraction from the liver of piglets. The control group (C) were fed with the basal diet. The mycotoxins experimental group were fed with the basal diet artificially contaminated with AFB1 and OTA (M group). Top 20 KEGG Pathways sorted by lowest *p* value are shown.

**Figure 3:**
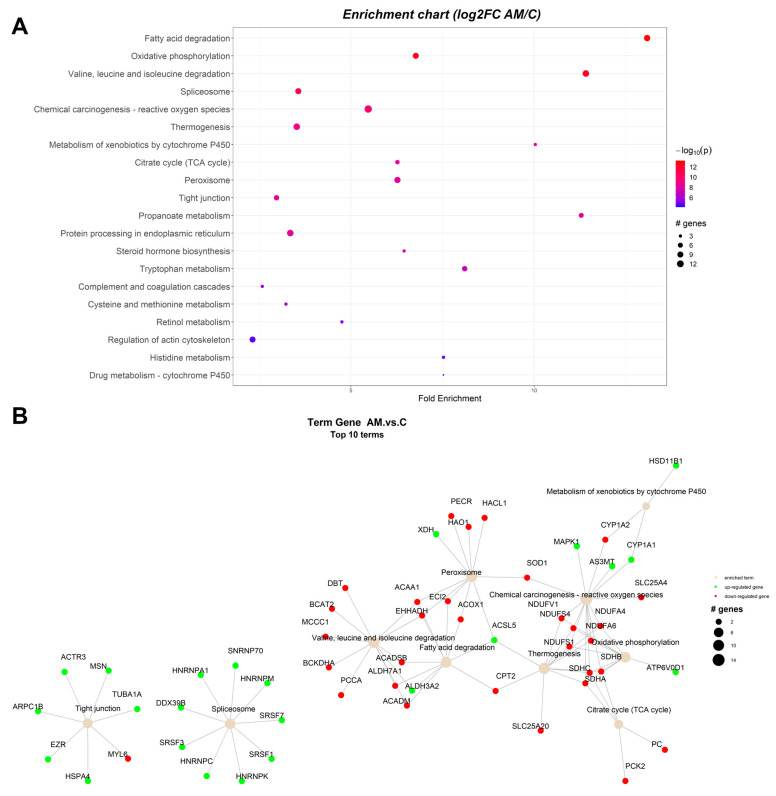
Enrichment chart (**A**) and term gene graph (**B**) from log2FC (AM/C) data for the microsomal fraction of the liver of piglets. The control group piglets (C) were fed with the basal diet. The members of the mycotoxins experimental group were fed with the basal diet containing the mixture (1:1) of grapeseed and sea buckthorn meal and contaminated with the mix of AFB1 and OTA (AM group). Top 20 KEGG Pathways sorted by lowest *p* value are shown.

The protein-protein interaction network (PIN) was constructed in R statistical software package, using the PathfindR module and proteins database for *Sus scrofa* from STRING. The results indicated that 186 unique gene products have been found to be expressed significantly differently in the studied conditions. Enrichment charts containing the top 20 pathways and Term gene graphs containing the top 10 pathways (Figure 1, Figure 2 and Figure 3) were generated with PathfindR (version 1.6.4). The relative expression of each gene product from each experimental group (A-antioxidants treated, M-mycotoxins treated, AM-mycotoxins, and antioxidants treated) reported to control group (C) is shown on KEGG pathways generated by the Pathview R module (version 1.38.0).

#### 2.1.1. Proteomic Changes Associated with Dietary Byproduct Antioxidants Administration

The present study conducted network analyses to rank pathways between the antioxidants (A) and control (C) groups, which are depicted in Figure 1A. The identified proteins were linked with biological function terms, thereby enabling the investigation of multiple terms (top 10) associated with the significant proteins, as shown in Figure 1B. Up-regulated proteins were highlighted in green, while down-regulated ones were shown in red. The node size of the enriched term corresponds to the number of input proteins implicated in the pathway. For a comprehensive summary of all ranked pathways, please refer to Table 1. Pathway analysis between antioxidants (A) and control (C) groups showed changes in the expression level of various proteins involved in multiple metabolic pathways, including fatty acid degradation, oxidative phosphorylation, amino acid degradation, ribosome biogenesis, citric acid cycle, chemical carcinogenesis, thermogenesis, spliceosome, butanoate metabolism, propanoate metabolism, protein processing, and others (Table 1).

The potential involvement of ALDH3A2 in these pathways is accentuated by the up-regulation of this enzyme which participates in the metabolism of arginine and proline, oxidative phosphorylation, and the breakdown of fatty acids. Conversely, the down-regulation of proteins that are implicated in the breakdown of fatty acids, such as ACAT2, ACAA1, EHHADH, ACOX1, ACADM, and others, suggests that they may have a reduced impact on the degradation process.

Regarding the oxidative phosphorylation pathway, the up-regulation of ATP6V0D1 and the down-regulation of various NADH-ubiquinone oxidoreductase subunits (NDUFS1, NDUFS4, NDUFV1, NDUFA4, NDUFA6, NDUFA9, NDUFB7, NDUFB10) indicate a shift in the energy-producing machinery. This is further supported by the up-regulation of multiple subunits of succinate dehydrogenase (SDHA, SDHB, SDHC) in the citric acid cycle.

The up-regulation of some ribosomal proteins (RPS7, RPS19, RPL29) and the down-regulation of other ribosomal proteins (RPS3, RPS3A, RPS5, RPS13, and RPL27A) implicated in the ribosome biogenesis pathway point to a shift in the relative proportions of ribosomal subunits; therefore, the effectiveness of protein synthesis might be affected.

Our data also show that proteins involved in lysosome dependent pathways (ATP6V0D1, IGF2R, CLTC, AP1G1, AP1M1) and endoplasmic reticulum dependent pathways (SEC63, RRBP1, HYOU1, DNAJB11, HSP90B1, PRK-CSH, CANX, LMAN2, LMAN1, SEC13, SEC31A, PDIA4, ERP29, UBXN4, CAPN1) are up-regulated.

#### 2.1.2. Proteomic Changes in Pigs’ Liver Fed with Mycotoxins Contaminated Diet

Network analysis for ranked pathway analysis between the mycotoxins (M) group and the control (C) group are shown in Figure 2A, and the links between identified proteins and biological terms based on the node size of the corresponding enriched terms are shown in Figure 2B. Ranked pathways resulted from pathfindR are available in Table 2.

The analysis of pathways between the group fed with the mycotoxins (M) diet artificially contaminated with AFB1 and the control (C) group revealed that the presence of both AFB1 and OTA had a significant impact on several metabolic pathways, such as propanoate metabolism, TCA cycle, oxidative phosphorylation, steroid biosynthesis, protein processing in endoplasmic reticulum, chemical carcinogenesis (reactive oxygen species), thermogenesis, peroxisome, fatty acid degradation, fatty acid biosynthesis, amino acid metabolism, PPAR signaling pathway, butanoate metabolism, pyruvate metabolism, retinol, ribosome, and lysosome, among others (as shown in Table 2). The proteins BCKDHA, BCKDHB, DBT, EHHADH, PCCA, SUCLG1, SUCLG2, and ALDH6A1 were down-regulated in propanoate metabolism, but ACSS2 and ECHDC1 were up-regulated. The citrate cycle (TCA cycle) was also impacted by down-regulation of ACO2, IDH2, SUCLG1, SU-CLG2, SDHA, SDHB, SDHC, PC, and PCK2.

Additionally, NDUFS1, NDUFS4, NDUFV1, NDUFA4, NDUFA9, SDHA, SDHB, and SDHC were down-regulated, which influenced oxidative phosphorylation. Also affected was steroid biosynthesis, where SQLE, LSS, TM7SF2, DHCR24, DHCR7 and LIPA were up-regulated.

Furthermore, HYOU1, DNAJC3, HSP90B1, GA-NAB, CANX, LMAN1, SEC13, SEC31A, BCAP31, and RAD23B were up-regulated, affecting protein processing in the endoplasmic reticulum, whereas RRBP1 and VCP were down-regulated. Our findings demonstrated that reactive oxygen species (ROS) also significantly influenced the chemical carcinogenesis pathway, up-regulating AS3MT and down-regulating NDUFV1, NDUFA4, NDUFA9, NDUFS1, NDUFS4, SDHA, SDHB, SDHC, SLC25A4, CAT, and EGFR.

While NDUFS1, NDUFS4, NDUFV1, NDUFA4, NDUFA9, SDHA, SDHB, SDHC, and SLC25A20 were down-regulated in the thermogenesis pathway, ACSL5 and ACSL1 were up-regulated. In peroxisomes ABCD3, ACSL5, ACSL1, and PRDX1 were up-regulated while EHHADH, ACAA1, IDH2, HAO1, and CAT were down-regulated. Additionally, fatty acid degradation was affected, with downregulation of ACAA1, EHHADH, ADH4, and ALDH7A1 and upregulation of ACSL5, ALDH3A2, and ACSL1.

The degradation pathway of leucine, valine, and iso-leucine was altered, ALDH3A2 and HMGCS1 being upregulated, while BCKDHA, BCKDHB, DBT, EHHADH, ACAA1, PCCA, ALDH6A1, ALDH7A1, MCCC1, and AUH were down-regulated.

#### 2.1.3. Proteomic Changes in Concomitant Administration of Antioxidants and Mycotoxins in Weaned Piglets’ Diet

Figure 3A displays network analysis for ranked pathway analysis between the group that was fed with the basal diet containing the mixture (1:1) of grapeseed and sea buckthorn meal and contaminated with the mix of AFB1 and OTA (AM group) and the control (C) group. According to Figure 3B, where up-regulated proteins are colored in green and down-regulated proteins are colored in red, the relationships between detected proteins and biological terms enable analysis of many terms (top 10) to which relevant proteins are associated. The number of input proteins involved in the route is represented by the node size of the enriched term. In Table 3 all ranking pathways from PathfindR output for AM vs. C comparation are listed.

Pathway analysis between AM and control (C) groups shows changes in the expression of various proteins involved in multiple metabolic pathways, including fatty acid degradation, oxidative phosphorylation, valine, leucine, and isoleucine degradation, chemical carcinogenesis-reactive oxygen species, thermogenesis, spliceosome, metabolism of xenobiotics by cytochrome P450, citrate cycle, peroxisome, propanoate metabolism, tight junction, steroid hormone biosynthesis, tryptophan metabolism, complement and coagulation cascades, cysteine and methionine metabolism, protein processing in endoplasmic reticulum, retinol metabolism, regulation of actin cytoskeleton, and lysine degradation (Table 3).

The findings from Figure 3 and Table 3 show that several metabolic pathways are impacted by the co-presence in the pig basal diet of the mixture (1:1) of grapeseed and sea buckthorn meal and the mix of AFB1 and OTA (AM group) versus C group.

Proteins involved in oxidative phosphorylation, such as ATP6V0D1, were also increased, as well as proteins involved in fatty acid degradation, such as ACSL5 and ALDH3A2. On the other hand, BCAT2 and BCKDHA, which are implicated in the degradation of valine, leucine, and isoleucine, were down-regulated.

Proteins involved in oxidative phosphorylation, such as NDUFV1 and NDUFA4, were down-regulated, whereas proteins involved in chemical carcinogenesis (through reactive oxygen species), such as AS3MT, CYP1A1 and MAPK1, were up-regulated.

### 2.2. Heatmap Analysis of Differentially Expressed Proteins of Hepatic Microsomal Fraction

**Figure 4:**
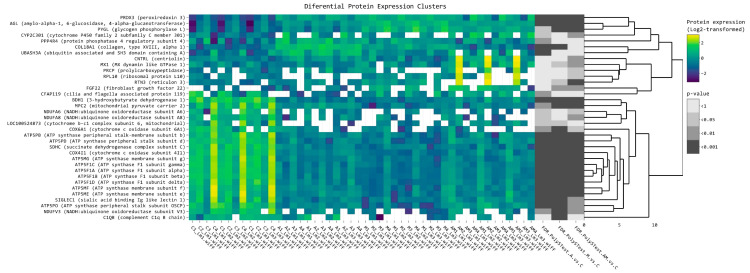
The most significantly changed protein expression levels clustering the protein affected by mycotoxins (M), antioxidants (A), and both (AM) diets versus the control diet in the microsomes extracted from liver tissues. Data was filtered for at least 2 folds change (log 2 threshold was set to exclude the interval −1, 1). Yellow color represents upregulation, while blue represents downregulation in experimental groups compared to control (C). From left to right, expression values (log2 transformed) for replicates (4 biological × 3 technical) are shown for the control (C) and 3 experimental groups (A, M, AM), followed by significance values of the respective comparisons to C.

Figure 4 shows the clustered heatmap of the 34 common differentially expressed proteins in all samples (4 biological × 3 technical replicates in each condition/diet). The heatmap shows two large clusters. The second and the larger cluster contains mostly OXPHOS mitochondrial complex subunits (*ATP synthase F1 subunit alpha*—*ATP5F1A*, *ATP synthase F1 subunit gamma*—*ATP5F1C*; *ATP synthase membrane subunit g*—*ATP5MG*; *cytochrome c oxidase subunit 4I1*—*COX4I1; succinate dehydrogenase complex subunit C*—*SDHC*; *ATP synthase peripheral stalk subunit d*—*ATP5PD*; *ATP synthase peripheral stalk-membrane subunit b*—*ATP5PB*; *cytochrome c oxidase subunit 6A1*—*COX6A1*; *cytochrome b-c1 complex subunit 6*, *mt*—*LOC100524873*; *NADH*:*ubiquinone oxidoreductase subunit A8*—*NDUFA8*; *NADH*:*ubiquinone oxidoreductase subunit A6*—*NDUFA6*), but also mitochondrial pyruvate carrier 2 (MPC2) and 3-hydroxybutyrate dehydrogenase 1 (BDH1), which are down-regulated in piglets fed with the mycotoxins contaminated diet (M) and in the antioxidants included group (A) and show a slightly ameliorated expression level in the mycotoxins and antioxidant (AM) group. The proteins in the first large cluster were split into smaller subclusters: PRDX3, AGL, and PYGL were slightly upregulated in group A, more up regulated in group M, and restored to almost group A level in the AM group. CYP2C301 was almost suppressed in groups A and M and restored in the AM group. Another subcluster showing significant variation between groups is formed by PPP4R4, COL18A1, and UBASH3A, which is up-regulated in group A, slightly up-regulated in group M, and restored to control levels in group AM. Another subgroup is formed of CNTR, MX1, PRCP, RPL10, RTN3, FGF22. These are down-regulated, even to an undetectable level in some group A and M samples, but up-regulated in AM. Notably one member of the AM group (AM2) shows a strongly up-regulated pattern for the components of this cluster.

In accordance with Figure 4, in Table 4 are presented log_2_ fold change (log_2_FC) data for each experimental group compared to the control group (A versus C, M versus C, and AM versus C) for corresponding proteins illustrated in the heatmap. The false discovery rate (FDR) was used to assess the significance of the results, and the lowest FDR value indicates the most significant result.

In A versus C comparison, peroxiredoxin 3 (PRDX3) showed a log_2_FC of 1.26 with an FDR of 2.55 × 10^−5^; the relative protein expression of PRDX3 was increased in condition A compared to the control condition. Protein phosphatase 4 regulatory subunit 4 (PPP4R4) also showed a significant increase in expression in condition A, with a log_2_FC of 1.61 and an FDR of 0.001337. On the other hand, 3-hydroxybutyrate dehydrogenase 1 (BDH1) showed a decrease in relative expression in condition A, with a log_2_FC of −1.14 and an FDR of 1.55 × 10^−10^.

Comparison between the M and C groups revealed that amylo-alpha-1, 6-glucosidase, and 4-alpha-glucanotransferase (AGL) showed the greatest increase in expression, with a log_2_FC of 1.41 and an FDR of 8.55 × 10^−9^. Also, MX dynamin-like GTPase 1 (MX1) showed an increase in expression, with a log_2_FC of 1.40425 and an FDR of 0.004935.

Similar to the A group compared to the control, in a comparison between the AM group and the C one, PRDX3 had the largest increase in relative protein expression with a log_2_FC of 1.19 and an FDR of 7.74 × 10^−5^. The relative protein expression of reticulon 3 (RTN3) increased significantly as well, with a log_2_FC of 1.48 and an FDR of 1.35 × 10^−6^.

### 2.3. Most significant Changes in Protein Expression

**Figure 5:**
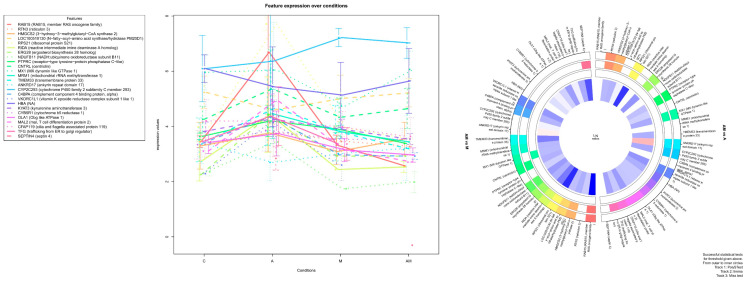
Expression Profiles for microsomes extracted from the liver tissues of piglets. Isolated effect of AFB1 and OTA (M) respective grapeseed and sea buckthorn meal (A) is shown by comparing the mixture (1:1) of grapeseed and sea buckthorn meal and mix of AFB1 and OTA (AM) data to antioxidants (A) respective mycotoxins (M) alone. Log2 fold change data between −1 and 1 was filtered out. PolyStest was used for statistical analysis. A filled box shows a protein with FDR < 0.01 for the corresponding comparison. The image was generated with PolyStest module in R software.

In order to investigate the effect of the mixture (1:1) of grapeseed and sea buckthorn meal (A group) and that of the mix of AFB1 and OTA, in Figure 5 are presented the log2FC data of the most significantly changed protein expression levels in the hepatic microsomal fraction of weaned piglets subjected to experimental diets, respectively, for 25 proteins significantlly changed in at least one of the AM versus A (mycotoxins effect) or AM versus M (antioxidants effect) comparisons. In accordance with Figure 5, in Table 5 are presented the log_2_FC data for the AM group compared to the A group, and in Table 6 are presented the log_2_FC data for the AM group compared to the M group, with corresponding FDR values for each protein represented in Expression Profiles graph (Figure 5).

#### 2.3.1. Impact of Mycotoxins

In Table 5 are shown the effects of mycotoxins on nine proteins, with different degrees of change in response to AFB1 and OTA contamination, sorted by FDR values (<0.01). Reticulon 3 (RTN3), 3-hydroxy-3-methylglutaryl-CoA synthase 2 (HMGCS2), ribosomal protein S21 (RPS21), ergosterol biosynthesis 28 homolog (ERG28), receptor-type tyrosine-protein phosphatase C-like (PTPRC), MX dynamin like GTPase 1 (MX1), cytochrome P450 family 2 subfamily C member 293 (CYP2C293), and vitamin K epoxide reductase complex subunit 1 like 1 (VKORC1L1) showed a significant increase in expression level. In contrast, ankyrin repeat domain 17 (ANKRD17) showed a significant decrease with a log2FC of −1.3 and an FDR of 0.007685.

#### 2.3.2. Impact of Antioxidants

Analyzing Table 6, it can be noted that the mixture of grapeseed and sea buckthorn meal significantly increased the fold change in the AM group versus the M group for 14 proteins, along with the false discovery rate (FDR) values. Out of these identified proteins, RAB15, member RAS oncogene family (RAB15) showed the highest Log_2_FC value of 4.66 with a significant FDR of 2.37 × 10^−6^. Ribosomal protein S21 (RPS21) also showed a high Log_2_FC value of 3.80 with a significant FDR of 0.000298. Reactive intermediate imine deaminase A homolog (RIDA) and transmembrane protein 33 (TMEM33) also showed significant Log_2_FC values of 2.45 and 2.37, respectively, with FDR values of 0.000316 and 0.007128. In contrast with mycotoxins isolated effect, for antioxidants isolated effect, RAB15, RIDA, NDUFB11, CNTRL, MRM1, TMEM33, and C4BPA are proteins significantly changed only in the AM group versus the M group (antioxidants effect).

## 3. Discussion

As far as we know, the present work represents the most comprehensive proteomics study to date investigating the effects of feed mycotoxins contamination and the potential of antioxidants to counteract these effects on a pig’s liver.

Using LC-MS, label-free, library-free, and unbiassed SWATH-MS data-independent acquisition (DIA) methods make our data reusable in future studies, and it can effortlessly be reanalyzed once proteome databases and protein-protein interactions knowledge and available software tools improve. Peptide deconvolution and identification with state-of-the-art neural networks and interference correction software (DIA-NN) [47] enables deep proteome coverage with single dimension LC separation.

In order to highlight the isolated and synergic effects of mycotoxins and antioxidants, statistical analysis was performed in PolyStest (Supplementary Material, Table S2). Datasets expression levels for each detected protein were compared as follows: mycotoxins (M) vs. control (C), antioxidants (A) vs. C, A + M (AM) vs. C, AM vs. A, AM vs. M.

The most statistically significant effect of OTA and AFB1 administered with a mixture of grapeseed and sea buckthorn meal (Table 5) was the overexpression of reticulon 3 (RTN3) involved in the regulation of the endoplasmic reticulum (ER) and Golgi apparatus shape and function. Previously, it was shown that another mycotoxin (T-2 toxin) led to chondrocyte apoptosis through the endoplasmic reticulum stress (ERS) pathway [48] or generally by activation of several caspases, including caspase-9, -8, and -3 through intrinsic and extrinsic pathways [49]. However, no evidence of any specific role of this protein in the liver was found in the literature, and KEGG has no link to it on any pathway that may apply to the liver in any species. A strong up-regulation in the AM group is shown in the sub-cluster formed by CNTR, MX1, PRCP, RPL10, RTN3, and FGF22. CNTRL is involved in the formation and maintenance of centrosomes and spindle poles, crucial structures in cell division (Figure 4). MX1 is a GTPase implicated in the regulation of membrane dynamics and has been implicated in the regulation of cell migration and immune response [50]. RPL10 is part of assembly and function of ribosomes and protein synthesis, while FGF22 plays a role in the regulation of cell growth, division, and differentiation [51,52,53]. Interestingly, our data show that exposure to OTA and AFB1 upregulated 3-hydroxy-3-methylglutaryl-CoA synthase 2 (HMGCS2), an enzyme involved in the biosynthesis of ketone bodies, a process that plays a crucial role in the regulation of energy metabolism increasing the intracellular ketone level and inhibiting cell proliferation [54,55].

**Figure A1:**
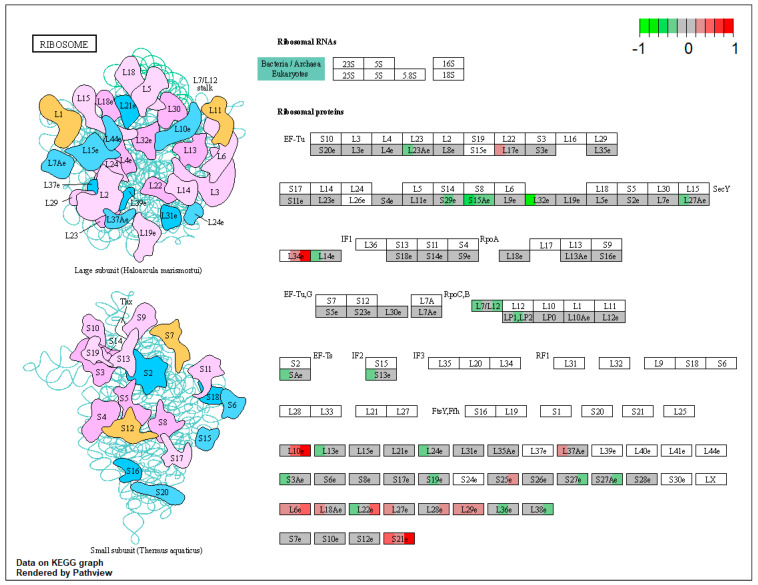
The KEGG ribosome pathway for the microsomal fraction of the liver. The color of the boxes represents the log_2_ fold change in the protein abundances, represented simultaneously for all three comparisons, on the left for A vs. C, in the middle for M vs. C and on the right for AM vs. C in the corresponding box for each protein.

Ribosomal component proteins were slightly upregulated, notably ribosomal protein S21 (RPS21) (Figure A1). Ergosterol biosynthesis 28 homolog (ERG28), previously known as contributing to cholesterol synthesis [56], is significantly overexpressed, while receptor-type tyrosine-protein phosphatase C-like (PTPRC) was overexpressed and has no documented effect in pig liver in the existing scientific literature. In humans, the homologous protein was identified as a target for hepatic carcinoma treatment [57]. Overexpression of MX dynamins like GTPase 1 (MX1) may indicate a hyperactivity in innate immunity response, as they are known to play a role in the antiviral defense [58].

Our data show that exposure to OTA and AFB1 in the M group down-regulated ankyrin repeat domain 17 (ANKRD17), which in previous studies was shown to be involved in liver development in mice [59], but it was also speculated that it has a function in cell cycle regulation and DNA replication [60].

The up-regulation of CYP2C293 (Table 5) is not a surprise, as this is known for its role in xenobiotics metabolism [61,62].

Vitamin K epoxide reductase complex subunit 1 like 1 (VKORC1L1), which regulates vitamin K metabolism, can support γ-carboxylation in vivo [63]. This enzyme is present in microsomal fraction of hepatocytes [64].

Receptor-type tyrosine-protein phosphatase C-like (PTPRC), ankyrin repeat domain 17 (ANKRD17), and CYP2C293 are proteins significantly changed only in AM group versus A group (mycotoxins effect).

Interestingly, the protein expression of several key proteins was affected mainly or only by the mycotoxins contaminated diet and restored after antioxidants addition (AM group) in feed.

**Figure A2:**
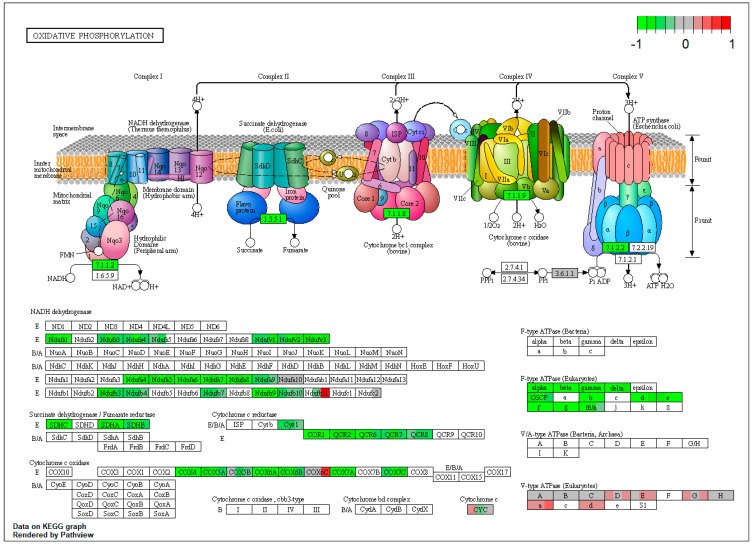
The KEGG oxidative phosphorylation pathway for the microsomal fraction of the liver. The color of the boxes represents the log_2_ fold change in the protein abundances, represented simultaneously for all three comparisons, on the left for A vs. C, in the middle for M vs. C and on the right for AM vs. C in the corresponding box for each protein.

Previously, direct effects of animal feed on oxidative phosphorylation (OXPHOS) pathway have been shown [65,66,67]. Our data clearly shows significant differences in the protein expression of the mitochondrial electron transport chain between the experimental diets used. The mitochondrial electron transport chain consists of four multi-enzyme complexes which oxidize NADH by Complex I or reduce FADH_2_ by Complex II and electron transfer to Complex III and Complex IV. As a result, an electrochemical proton gradient is created, driving complex V to produce adenosine triphosphate (ATP) in a process known as oxidative phosphorylation [68]. The coordinated action of numerous accessory and catalytic subunits that make up these complexes (as shown in Figure A2), determines how effective this process will be. Therefore, modifications in how these subunits are expressed may have a strong effect on the electron transport chain. This is the case in our proteomics data: hierarchical clustering based on the experimental group and fold change compared to the control group depicts OXPHOS proteins forming groups with significant and corelated variation patterns (Figure 4).

**Figure A3:**
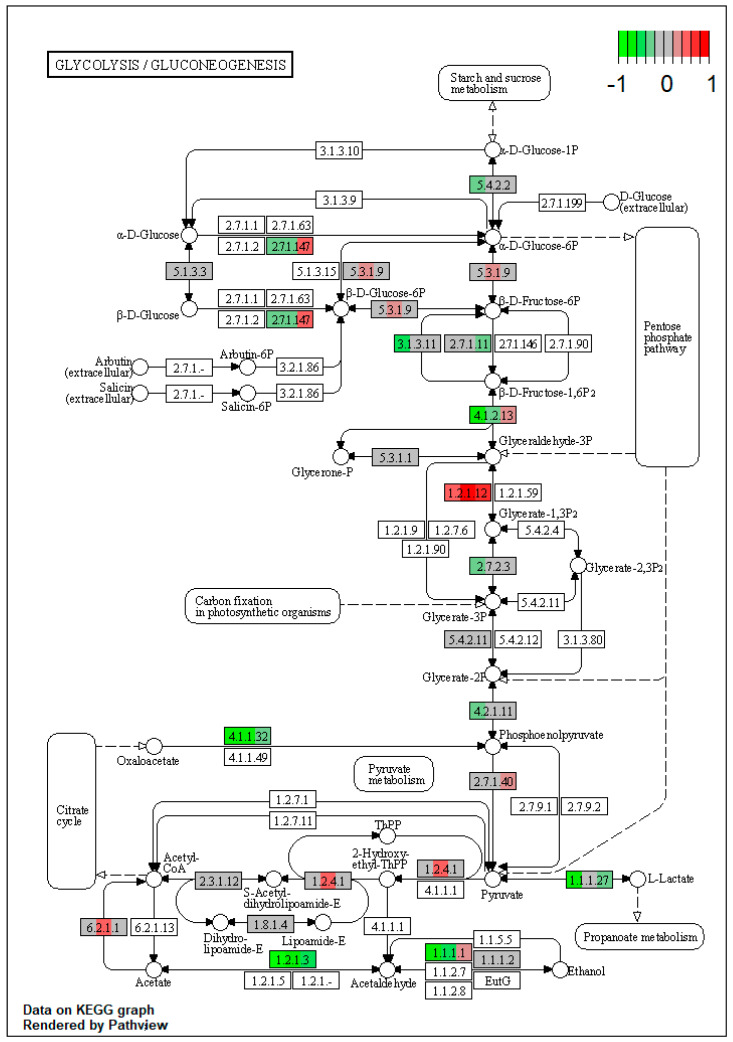
The KEGG glycolysis pathway for the microsomal fraction of the liver. The color of the boxes represents the log_2_ fold change in the protein abundances, represented simultaneously for all three comparisons, on the left for A vs. C, in the middle for M vs. C and on the right for AM vs. C in the corresponding box for each protein.

**Figure A4:**
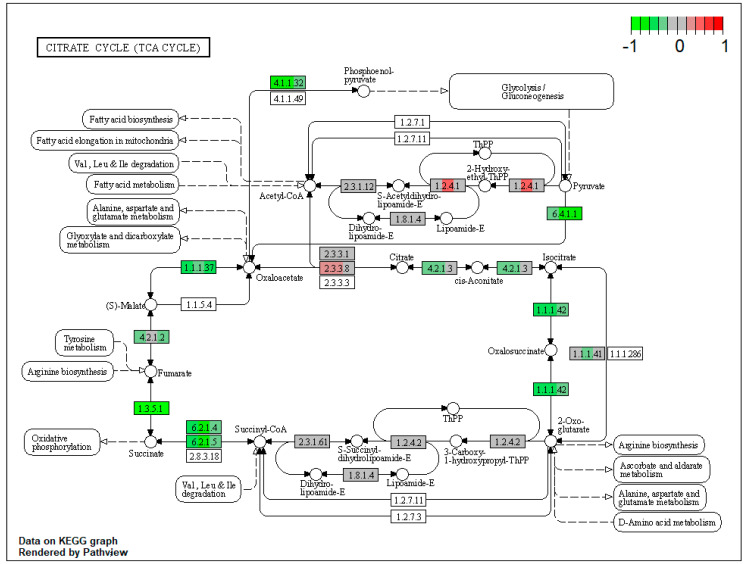
The KEGG TCA cycle pathway for the microsomal fraction of the liver. The color of the boxes represents the log_2_ fold change in the protein abundances, represented simultaneously for all three comparisons, on the left for A vs. C, in the middle for M vs. C and on the right for AM vs. C in the corresponding box for each protein.

**Figure A5:**
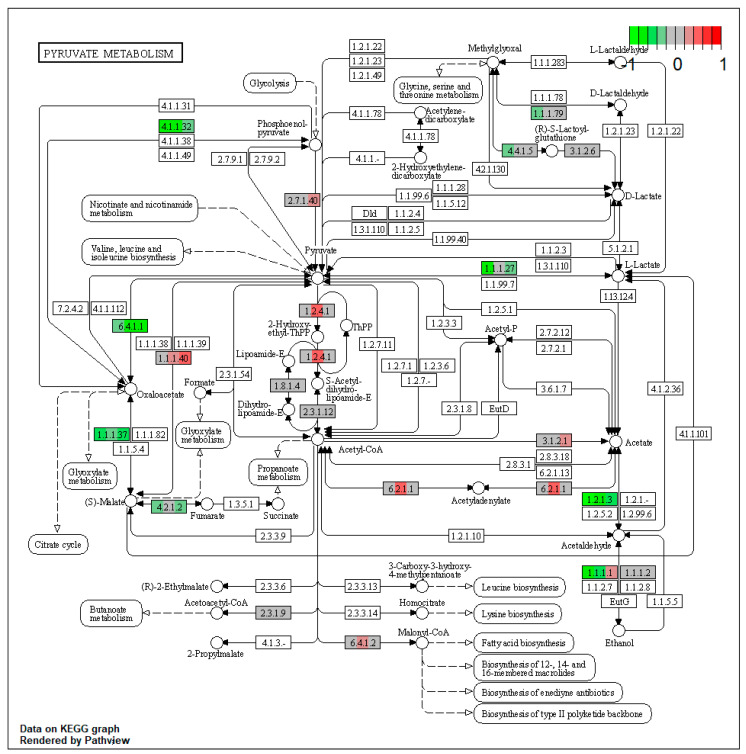
Pyruvate metabolism. The KEGG pyruvate metabolism pathway for the microsomal fraction of the liver. The color of the boxes represents the log_2_ fold change in the protein abundances, represented simultaneously for all three comparisons, on the left for A vs. C, in the middle for M vs. C and on the right for AM vs. C in the corresponding box for each protein.

NDUFA6 and NDUFA8 are subunits of Complex I of the respiratory chain, involved in the transfer of electrons from NADH to ubiquinone, an important step in oxidative phosphorylation. Complex I down-regulation stimulates glucose metabolism (Figure A3) and is associated with changes in mitochondrial morphology and slowdown in the TCA cycle rate (Figure A4) [69], which are linked with pyruvate metabolism (Figure A5) in the process of energy metabolism of the cell [70]. The control of the TCA cycle and its ongoing interaction with respiratory complexes are essential for maintaining the stability of the cells [71].

SDHC is a subunit of Complex II and a more pronounced down-regulation of proteins expression of this complex in the group where the basal diet is contaminated with AFB1 and OTA (M group) compared with A and AM groups was observed. This significant down-regulation could suggest a lower ability of Complex II to produce ROS [72] and trigger apoptosis pathway in response to pro-apoptotic signals. Moreover, down-regulation of expression and activity of Complex II could be also a consequence of the general shift from OXPHOS to glycolysis [73], which correlates with our data from the glycolysis pathway (Figure A3), where an upregulation of glucose-6-phosphate isomerase (GPI—EC 5.3.1.9) and glyceraldehyde 3-phosphate dehydrogenase (GAPDH—EC 1.2.1.12) protein expression levels was observed in the M compared to the C group and restored to control after antioxidants addition.

**Figure A6:**
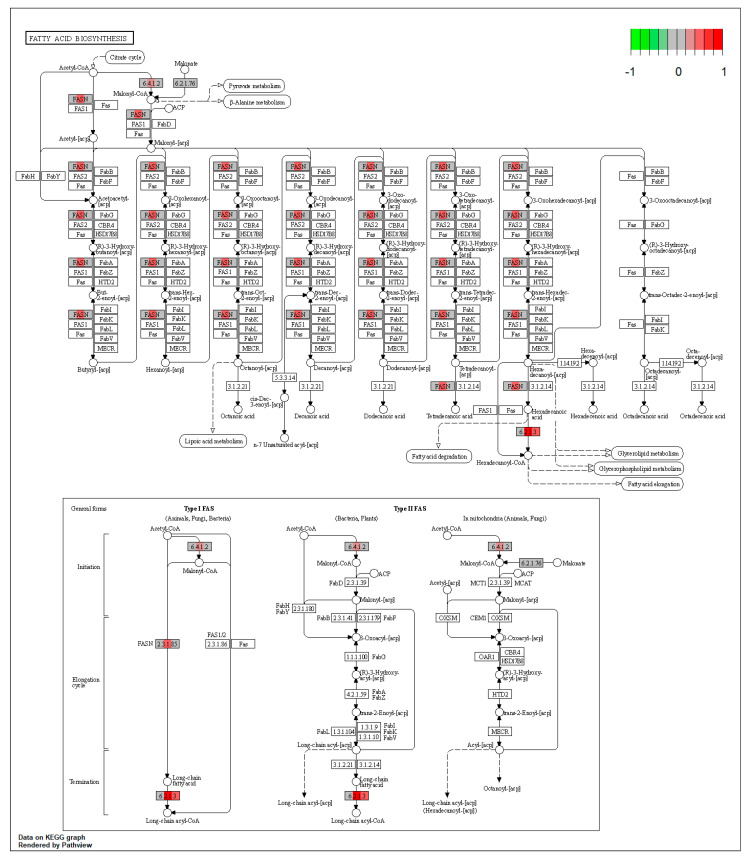
The KEGG fatty acid biosynthesis pathway for the microsomal fraction of the liver. The color of the boxes represents the log_2_ fold change in the protein abundances, represented simultaneously for all three comparisons, on the left for A vs. C, in the middle for M vs. C and on the right for AM vs. C in the corresponding box for each protein.

**Figure A7:**
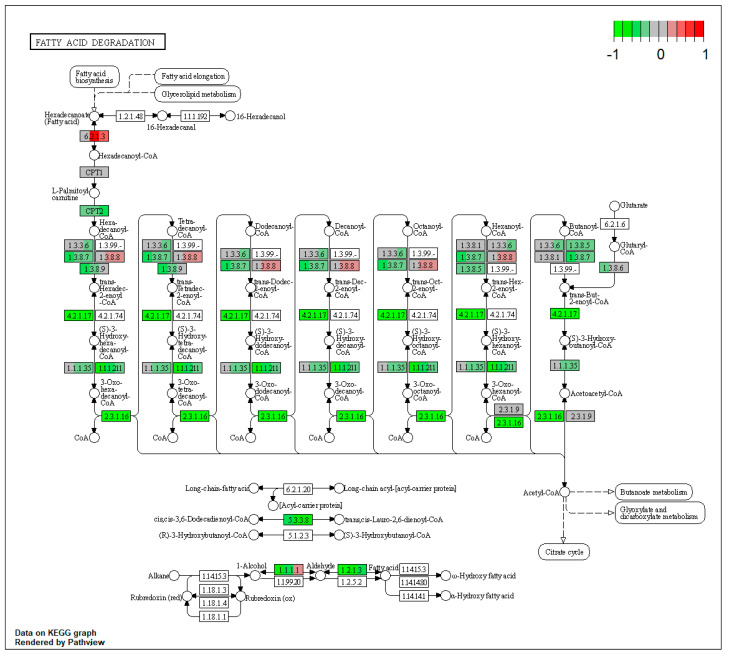
The KEGG fatty acid degradation pathway for the microsomal fraction of the liver. The color of the boxes represents the log_2_ fold change in the protein abundances, represented simultaneously for all three comparisons, on the left for A vs. C, in the middle for M vs. C and on the right for AM vs. C in the corresponding box for each protein.

**Figure A8:**
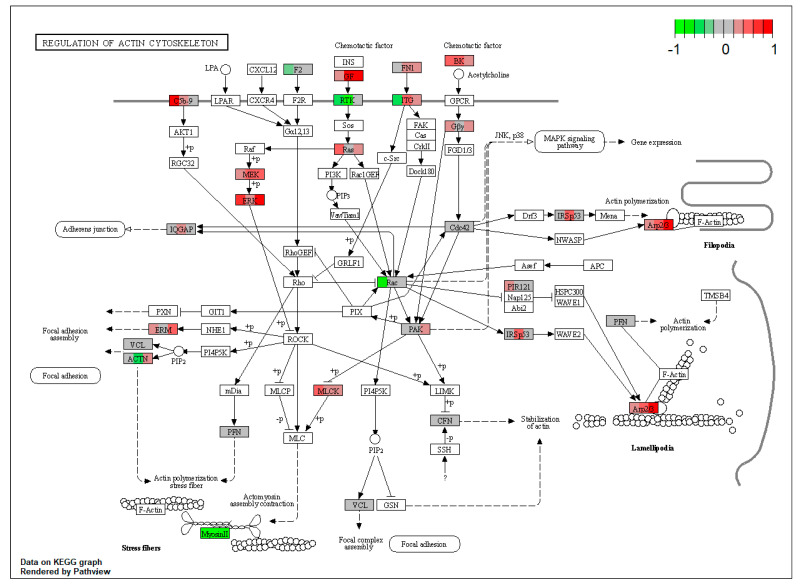
The KEGG pathway of regulation of actin cytoskeleton from the microsomal fraction of the liver. The color of the boxes represents the log_2_ fold change in the protein abundances, represented simultaneously for all three comparisons, on the left for A vs. C, in the middle for M vs. C and on the right for AM vs. C in the corresponding box for each protein.

**Figure A9:**
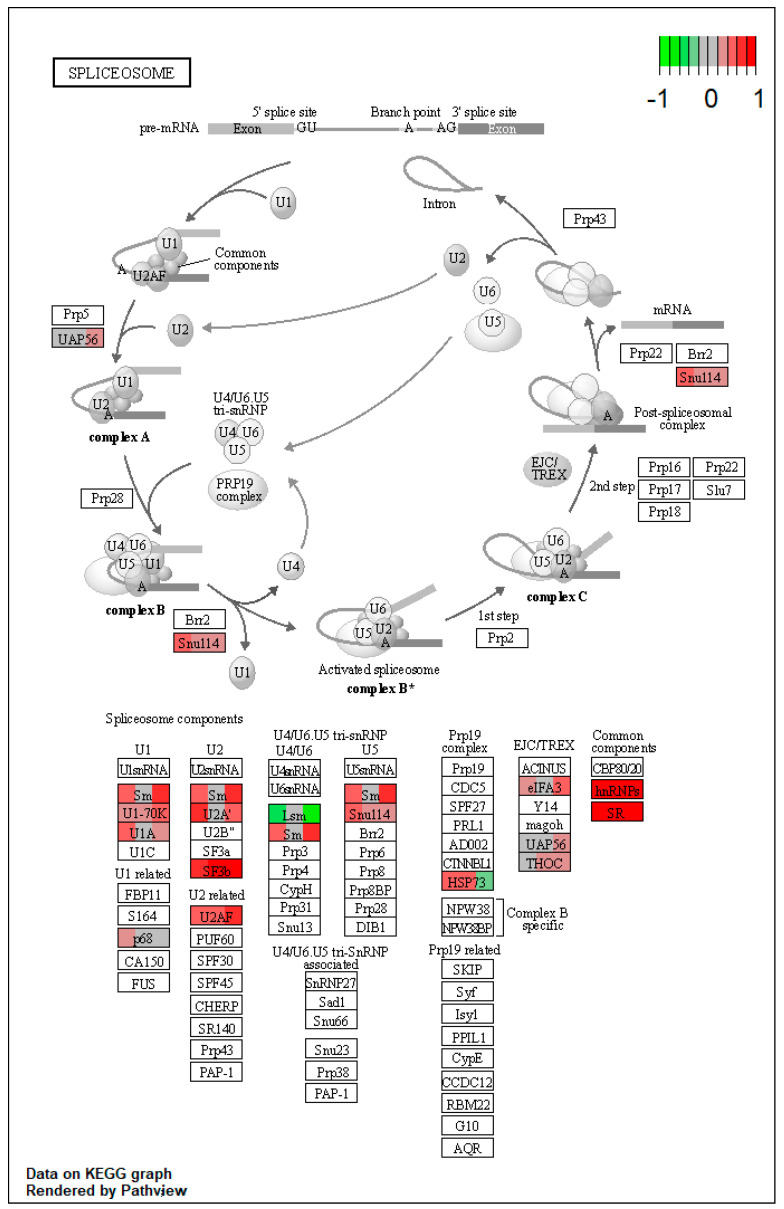
The KEGG spliceosome pathway for the microsomal fraction of the liver. The color of the boxes represents the log_2_ fold change in the protein abundances, represented simultaneously for all three comparisons, on the left for A vs. C, in the middle for M vs. C and on the right for AM vs. C in the corresponding box for each protein.

**Figure A10:**
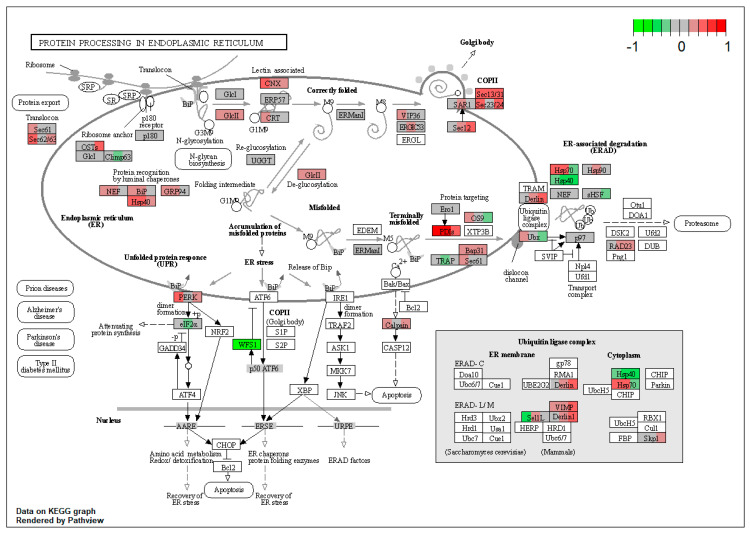
The KEGG protein processing in endoplasmic reticulum pathway for the microsomal fraction of the liver. The color of the boxes represents the log_2_ fold change in the protein abundances, represented simultaneously for all three comparisons, on the left for A vs. C, in the middle for M vs. C and on the right for AM vs. C in the corresponding box for each protein.

**Figure A11:**
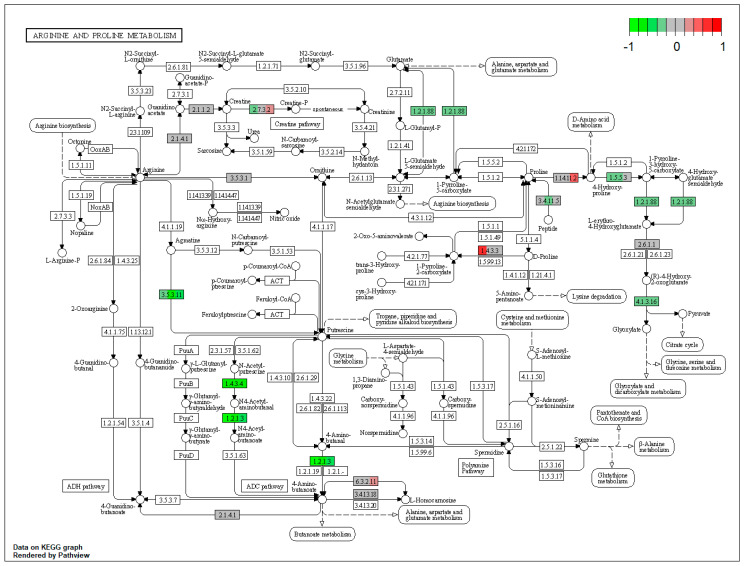
The KEGG arginine and proline metabolism pathway for the microsomal fraction of the liver. The color of the boxes represents the log_2_ fold change in the protein abundances, represented simultaneously for all three comparisons, on the left for A vs. C, in the middle for M vs. C and on the right for AM vs. C in the corresponding box for each protein.

**Figure A12:**
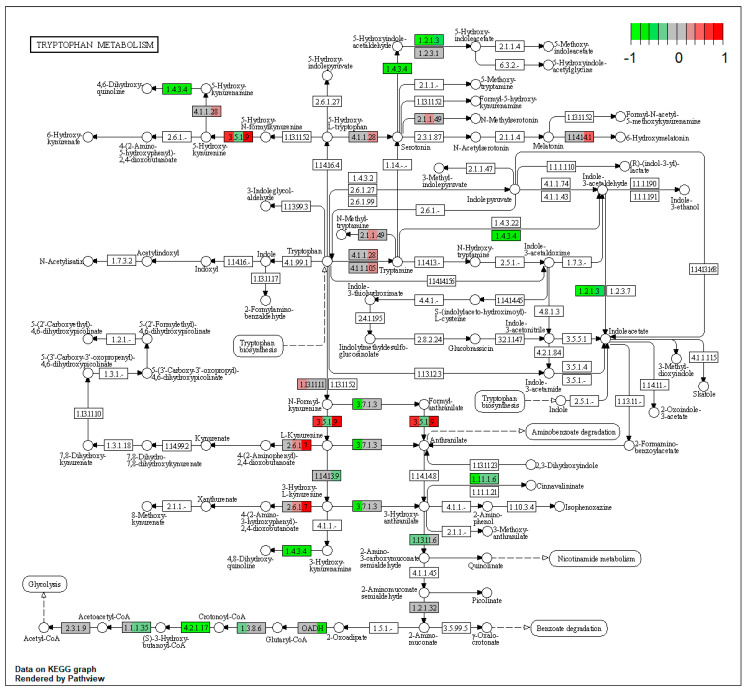
The KEGG tryptophan metabolism pathway for the microsomal fraction of the liver. The color of the boxes represents the log_2_ fold change in the protein abundances, represented simultaneously for all three comparisons, on the left for A vs. C, in the middle for M vs. C and on the right for AM vs. C in the corresponding box for each protein.

**Figure A13:**
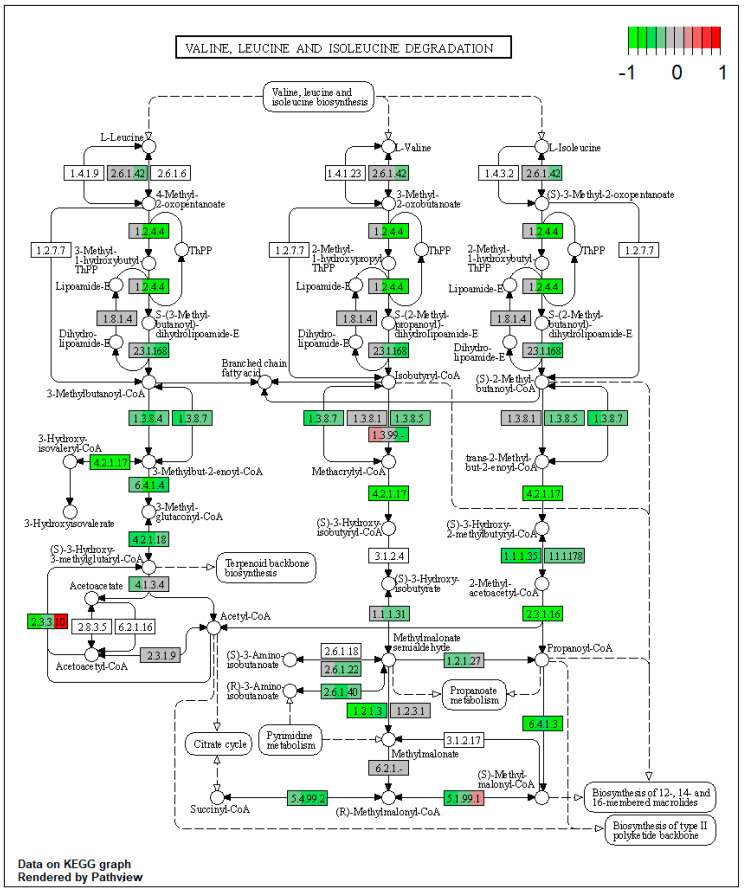
The KEGG valine, leucine, and isoleucine degradation pathway for the microsomal fraction of the liver. The color of the boxes represents the log_2_ fold change in the protein abundances, represented simultaneously for all three comparisons, on the left for A vs. C, in the middle for M vs. C and on the right for AM vs. C in the corresponding box for each protein.

**Figure A14:**
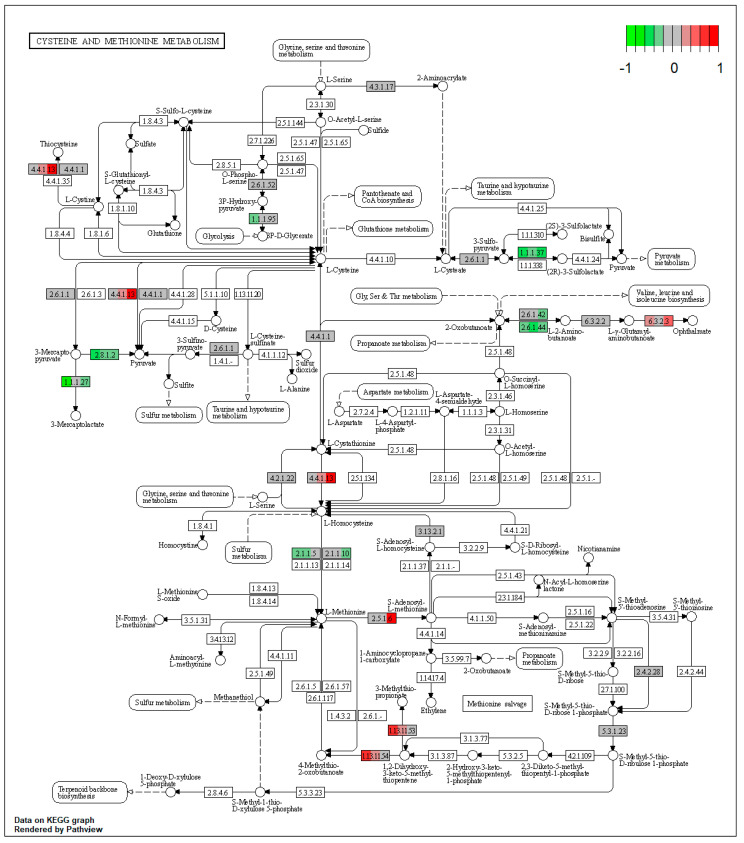
The KEGG cysteine and methionine metabolism pathway for the microsomal fraction of the liver. The color of the boxes represents the log_2_ fold change in the protein abundances, represented simultaneously for all three comparisons, on the left for A vs. C, in the middle for M vs. C and on the right for AM vs. C in the corresponding box for each protein.

**Figure A15:**
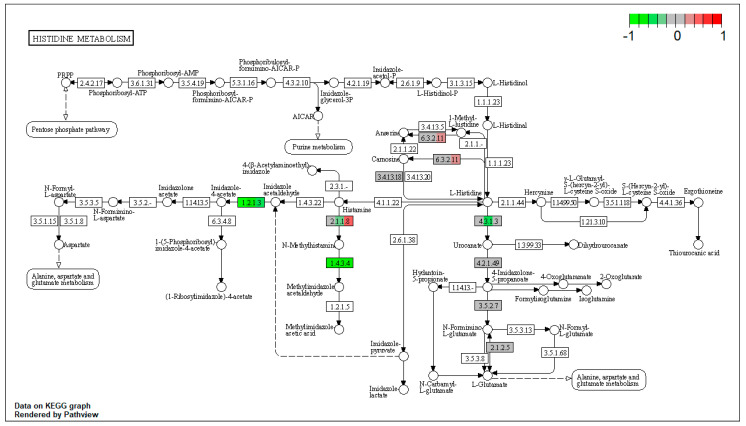
The KEGG histidine metabolism pathway for the microsomal fraction of the liver. The color of the boxes represents the log_2_ fold change in the protein abundances, represented simultaneously for all three comparisons, on the left for A vs. C, in the middle for M vs. C and on the right for AM vs. C in the corresponding box for each protein.

LOC100524873 is a part of the ubiquinol-cytochrome c reductase complex, also called Complex III, and plays an important role in maintaining the structural and functional integrity of the mitochondria; its down-regulation led to increased levels of ROS [74,75] and is associated with alterations in fatty acid metabolism (Figure A6 and Figure A7), regulation of actin cytoskeleton (Figure A8), regulation of gene expression by spliceosomes (Figure A9), membrane trafficking (Figure A10), and alterations in amino acids metabolism (Figure A11, Figure A12, Figure A13, Figure A14 and Figure A15) [76].

Supporting the validity of our results, expression of COX6A1 and COX4I1, subunits of complex IV, was down-regulated. In the liver, mitochondrial complex IV down-regulation has both biochemical and functional consequences because it is required for cellular redox status maintenance [77], which correlates with our data by which Complex II is also down-regulated. Complex II is the only one that participates in both the TCA cycle and the electron transport chain [78]. A suppressed Complex II mobilizes amino acids metabolism [79]. Further pathway enrichment analysis shows that arginine, proline (Figure A11), and tryptophan metabolism (Figure A12), valine, leucine, and isoleucine degradation (Figure A13), cysteine and methionine metabolism (Figure A14), and histidine metabolism (Figure A15) pathways were the most affected. Interestingly, a link between amino acids pathways and TCA cycle has been previously shown [80].

As shown in Figure A11, arginine is regenerated by arginosuccinate lyase (ASL—EC 4.3.2.1) and argininosuccinate synthase 1 (ASS1—EC 6.3.4.5), thus protein expression remains unchanged. Complex II activates the arginine biosynthesis pathway, which maintains fumarate levels and preserves TCA metabolic activity in vivo [78]. Amino acids metabolism has a significant role in determining the nutritional value of pork meat [81].

ATP5F1C, ATP5MG, ATP5PD, and ATP5PB are subunits of the F1 component of ATPase (Complex V) involved in ATP generation. An enhanced aerobic glycolysis led to impaired oxidative phosphorylation [82] and, therefore, ATP generation and Complex V suppression, which is confirmed by our data where the protein expression for the identified subunits is down-regulated. Up-regulation of AS3MT, CYP1A1, and MAPK1 can result in higher oxidative stress, leading to potential harm to the animals (Figure 4). The V type ATPase couples ATP hydrolysis to active proton transport across membranes and its protein expression and activity is interconnected with glycolysis (Figure A3) [83]. ATP6V1E1, ATP6V0A1, and ATP6V0D1 (Figure A2) protein expressions are up-regulated in the experimental group fed with the diet contaminated with AFB1 and OTA (M group) compared with the A and AM groups, and is correlated with the expression of GPI (EC 5.3.1.9), GAPDH (EC 1.2.1.12), PDHA1 (EC 1.2.4.1), and ACSS2 (EC 6.2.1.1) belonging to glycolysis and gluconeogenesis metabolism (Figure A3).

**Figure A16:**
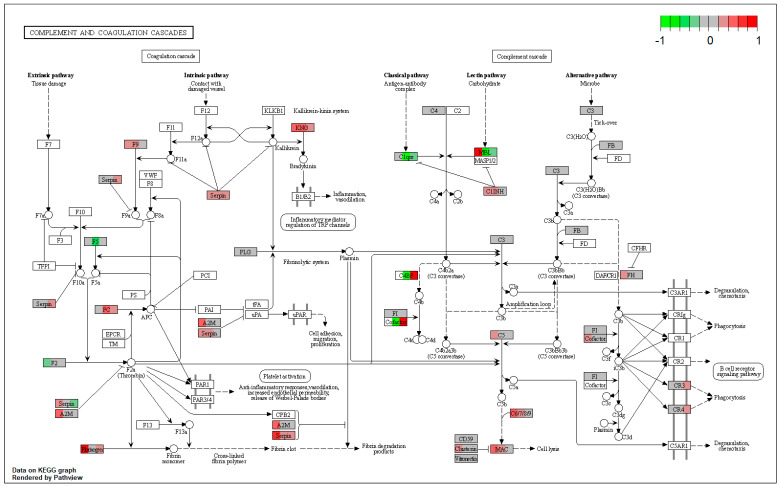
The KEGG complement and coagulation cascades pathway for the microsomal fraction of the liver. The color of the boxes represents the log_2_ fold change in the protein abundances, represented simultaneously for all three comparisons, on the left for A vs. C, in the middle for M vs. C and on the right for AM vs. C in the corresponding box for each protein.

Next, we analyzed the effect of experimental diets on β-oxidation of fatty acids and it seems that mycotoxins exposure decreased mitochondrial function and this metabolic pathway (Figure A7). Moreover, FASN protein expression is up-regulated (Figure A6), which is associated with a decrease in the activation of energy-sensing pathways and the accumulation of lipid droplets [84]. Also, immunotoxic effects due to OTA presence in the diet [85] of the M group could be due to its involvement in a complement and coagulation cascade (Figure A16) through MBL1, MBL2, C4BPA, C4A, F9, and F5. Co-administration of antioxidants (AM group) ameliorated most of the observed immunotoxic effects of mycotoxins.

**Figure A17:**
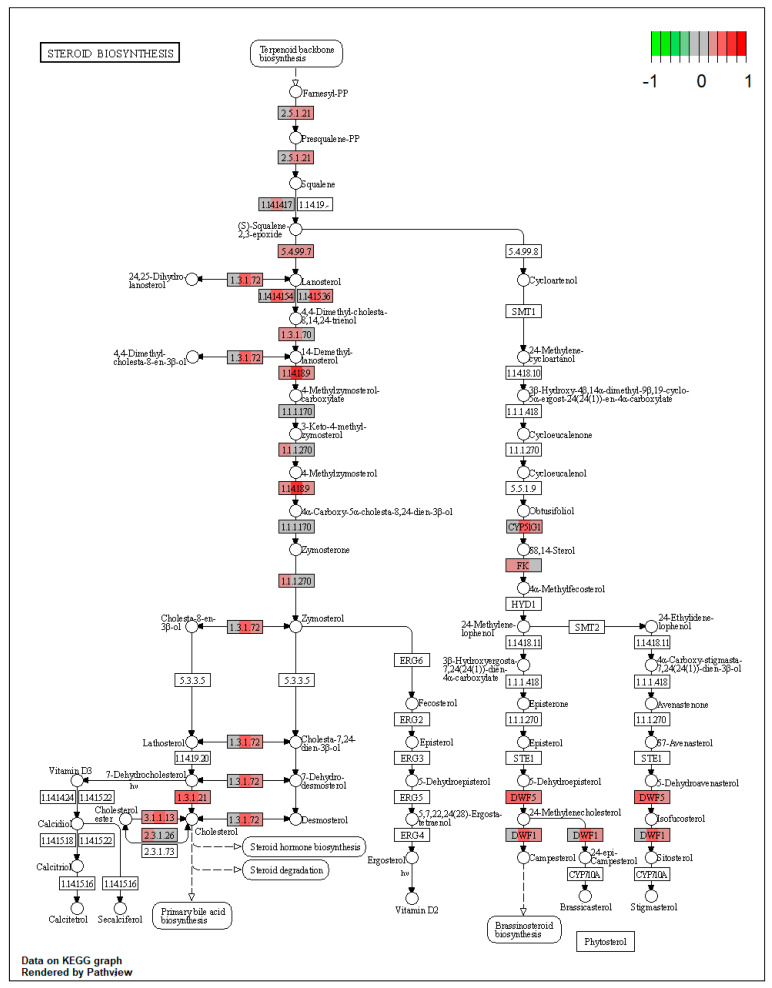
The KEGG steroid metabolism pathway for the microsomal fraction of the liver. The color of the boxes represents the log_2_ fold change in the protein abundances, represented simultaneously for all three comparisons, on the left for A vs. C, in the middle for M vs. C and on the right for AM vs. C in the corresponding box for each protein.

**Figure A18:**
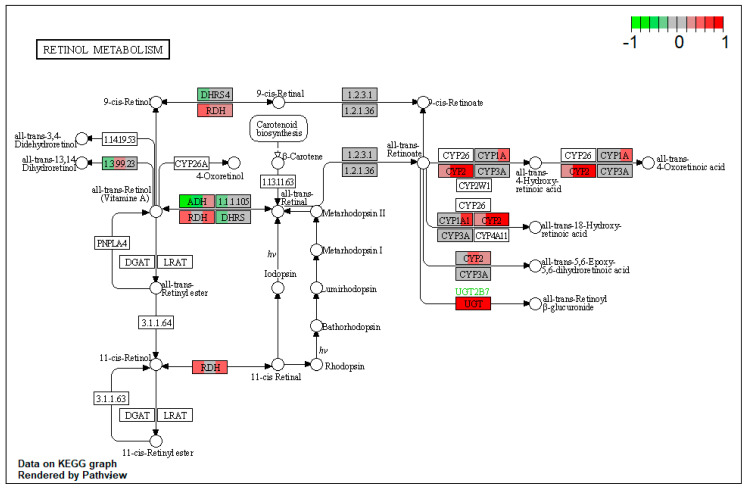
The KEGG retinol metabolism pathway for the microsomal fraction of the liver. The color of the boxes represents the log_2_ fold change in the protein abundances, represented simultaneously for all three comparisons, on the left for A vs. C, in the middle for M vs. C and on the right for AM vs. C in the corresponding box for each protein.

Steroid hormones are crucial for lipid metabolism, and hepatocellular injury has been linked to the down-regulation of steroid metabolism (Figure A17) [86]. Our results showed a general up-regulation of steroid biosynthesis, which may be caused by retinol metabolism (Figure A18) [87].

**Figure A19:**
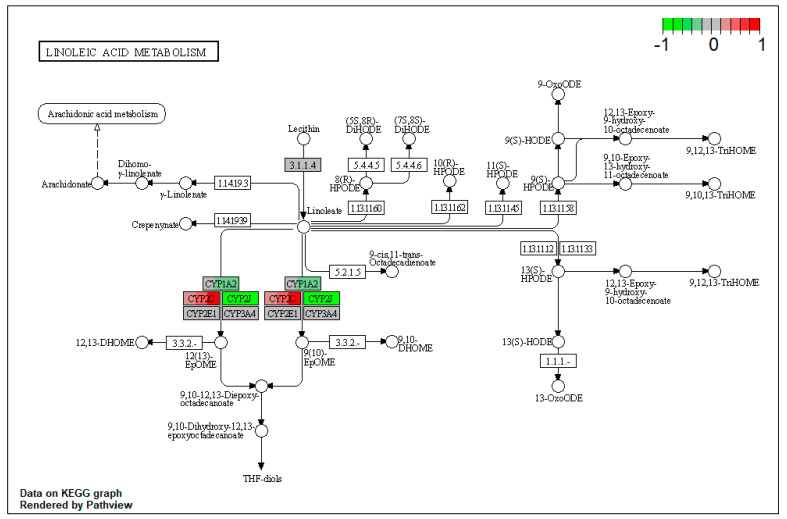
The KEGG linoleic acid metabolism pathway for the microsomal fraction of the liver. The color of the boxes represents the log_2_ fold change in the protein abundances, represented simultaneously for all three comparisons, on the left for A vs. C, in the middle for M vs. C and on the right for AM vs. C in the corresponding box for each protein.

Interestingly, significant changes in linoleic metabolism are observed (Figure A19). Linoleic acid (C18:2) level is considered an indicator of fatness in pigs. Animals require linoleic acid and α-linolenic acid as necessary fatty acids for maintaining growth, reproduction, and brain development. Because mammals are unable to produce omega fatty acids on their own, they must obtain these polyunsaturated fatty acids (PUFAs) from their diet [88,89]. Of all fatty acids, linoleic acid shows the greatest tissue response to diet quality [90]. The mixture of antioxidants used in this study had a higher content of α-linolenic acid in sea buckthorn meal, and the grape seed meal has a very high linoleic acid content [45].

**Figure A20:**
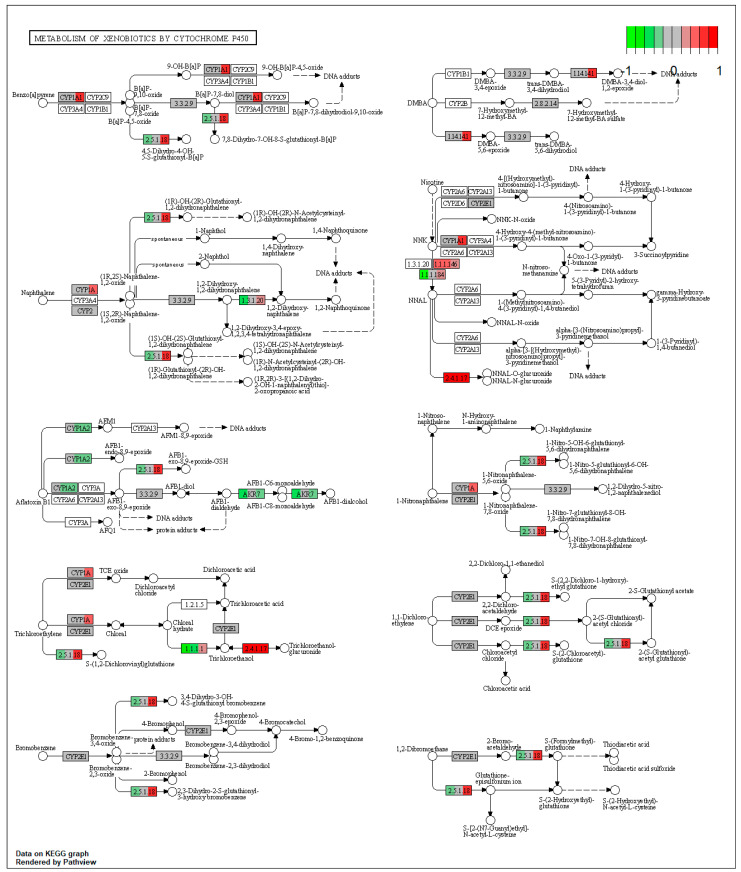
The KEGG metabolism of xenobiotics by cytochrome P450 pathway for the microsomal fraction of the liver. The color of the boxes represents the log_2_ fold change in the protein abundances, represented simultaneously for all three comparisons, on the left for A vs. C, in the middle for M vs. C and on the right for AM vs. C in the corresponding box for each protein.

**Figure A21:**
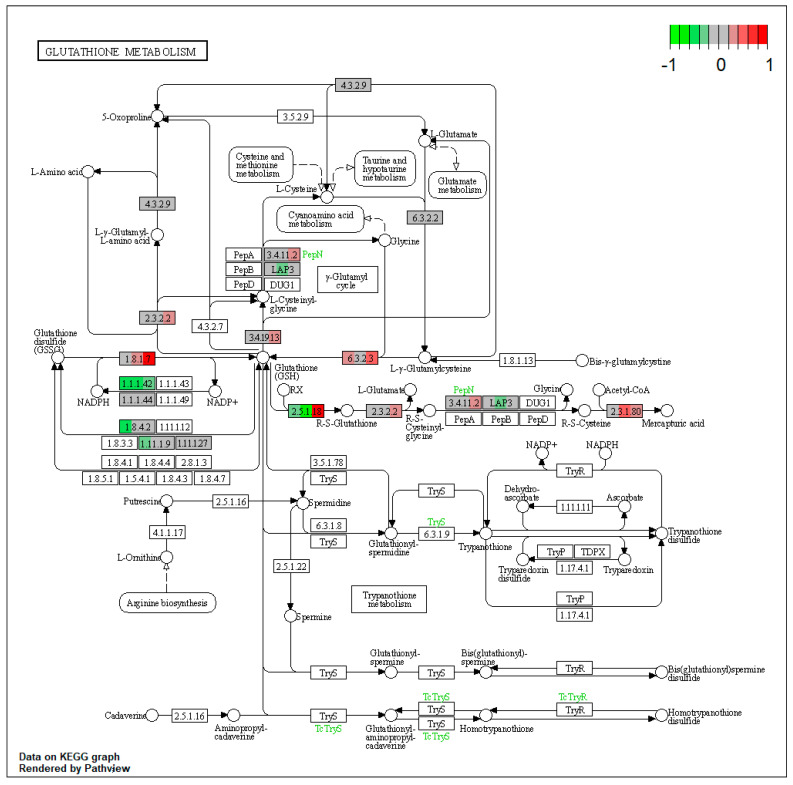
The KEGG glutathione metabolism pathway for the microsomal fraction of the liver. The color of the boxes represents the log_2_ fold change in the protein abundances, represented simultaneously for all three comparisons, on the left for A vs. C, in the middle for M vs. C and on the right for AM vs. C in the corresponding box for each protein.

**Figure A22:**
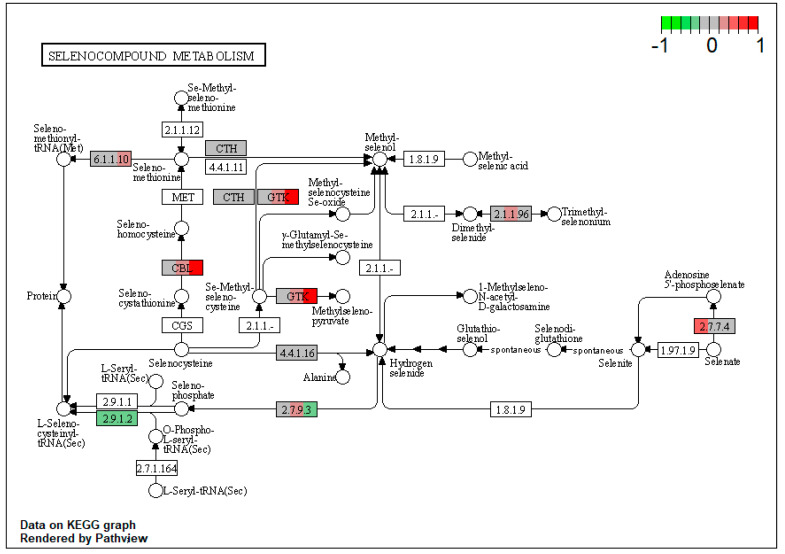
The KEGG selenocompound metabolism pathway for the microsomal fraction of the liver. The color of the boxes represents the log_2_ fold change in the protein abundances, represented simultaneously for all three comparisons, on the left for A vs. C, in the middle for M vs. C and on the right for AM vs. C in the corresponding box for each protein.

**Figure A23:**
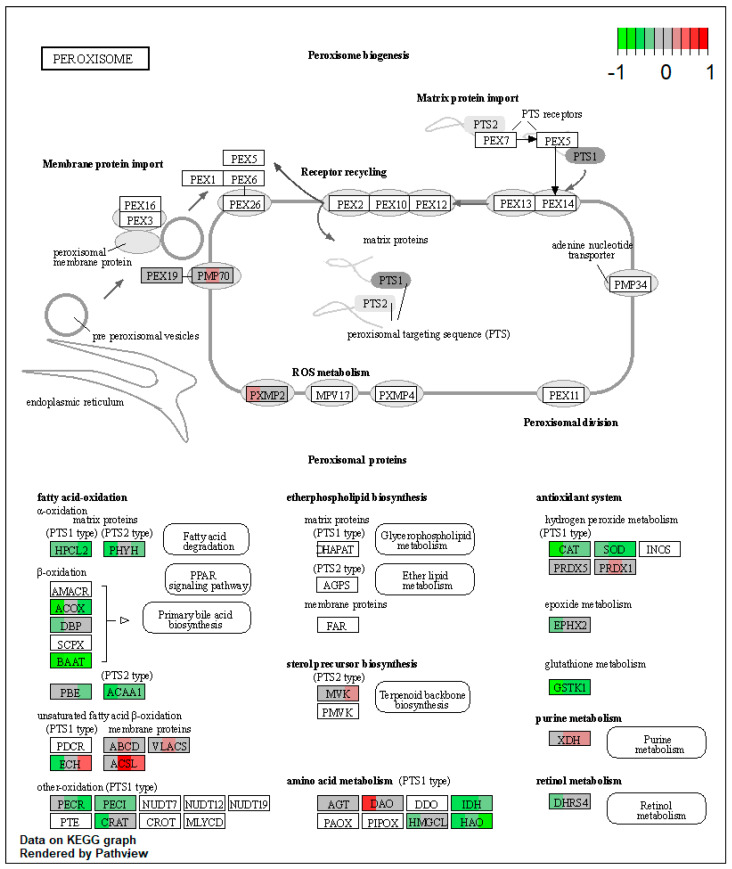
The KEGG peroxisome pathway for the microsomal fraction of the liver. The color of the boxes represents the log_2_ fold change in the protein abundances, represented simultaneously for all three comparisons, on the left for A vs. C, in the middle for M vs. C and on the right for AM vs. C in the corresponding box for each protein.

The observable modifications in the metabolism of xenobiotics can be attributed to the expression of cytochrome P450 (as illustrated in Figure A20). The principal enzyme involved in the processing of phase I metabolites, which is a result of the cytochrome P450 activity, is GSTA1 (EC 2.5.1.18). Following this, the resulting metabolites are then subjected to conjugation in phase II to enhance their hydrophilicity and facilitate excretion. GSTA1 enzyme expression is heightened when mycotoxins and antioxidants are present simultaneously (as depicted in Figure A21). INMT (EC 2.1.1.96) [91] is another phase II enzyme that participates in selenocompounds metabolism (as seen in Figure A22). In peroxisome metabolism (Figure A23) CAT is strongly repressed by antioxidants, while this effect is reduced by the presence of mycotoxins even in co-administration with antioxidants. SOD is also slightly repressed by antioxidants and more strongly repressed by the presence of mycotoxins, almost at the same level with or without antioxidants. SOD prevents cells from dying by dismutation of the superoxide byproducts of oxidative phosphorylation and converting them to hydrogen peroxide and molecular oxygen [92]. PRDX1 is not affected by antioxidants alone, it is overexpressed by when mycotoxins exposure occurs and turns back to normal level in antioxidants and mycotoxins co-administration, which suggests that PRDX1 has a role in ROS homeostasis [93].

In our prior investigation [46], it was shown that the introduction of a by-product blend of antioxidants upregulated CYP3A protein expression while reducing the specific enzyme activity in the liver of pigs. Nonetheless, when feed was contaminated with both AFB1 and OTA, the trend was the opposite. The present study’s results corroborated these findings, with CYP3A29 and CYP3A227 being up-regulated. Upon analysing the gene expression profiles, it was observed that the combination of grapeseed and sea buckthorn meal antioxidants mixture had the most statistically significant effects on genes such as RAB15, a member of the RAS oncogene family, reactive intermediate imine deaminase A homolog (RIDA), NADH: ubiquinone oxidoreductase subunit B11 (NDUFB11), centriolin (CNTRL), mitochondrial rRNA methyltransferase 1 (MRM1), transmembrane protein 33 (TMEM33), and complement component 4 binding protein, alpha (C4BPA), as presented in Table 6. RAB15 is a small GTPase that belongs to the RAS family of proteins and is involved in the intracellular vesicle and membrane trafficking [94]. RIDA has been implicated in various biological processes including DNA damage response, oxidative stress, and redox homeostasis [95]. NDUFB11 is a subunit of Complex I of the respiratory chain and is involved in the transfer of electrons from NADH to ubiquinone, an important step in oxidative phosphorylation [96]. MRM1 is involved in the modification of mitochondrial ribosomal RNA and plays a role in the regulation of mitochondrial protein synthesis [97]. TMEM33 is known to be implicated in membrane trafficking, cell proliferation, and immune response [98]. C4BP regulates complement activation by controlling C4b-mediated reactions and acts as an innate immune effector against influenza A virus [99], regulating viral replication levels by modulating viral entry. It looks like that in the presence of mycotoxins, antioxidants significantly affect the expression level of some proteins involved in endocytosis and membrane trafficking. They may protect cell membranes altogether from mycotoxin-induced damage. However, further studies are required to investigate the detailed molecular mechanism.

## 4. Conclusions

As far as we know, this is the first study analyzing the effects of feed mycotoxins contamination with AFB1 and OTA and the potential of a diet including by-product mixture of antioxidants to counteract these effects on weaned piglets’ liver, using an unbiased label-free, library-free, DIA mass spectrometry SWATH method. A total of 1878 quantifiable proteins were identified from microsomal fraction. The study confirmed the pathways involved in metabolism of xenobiotics by cytochrome P450, TCA cycle, glutathione, and oxidative phosphorylation pathways have been affected, as previously reported.

Pathway enrichment analysis of the differentially expressed proteins showed that fatty acid metabolism, steroid biosynthesis, regulation of actin cytoskeleton, regulation of gene expression by spliceosomes, membrane trafficking, peroxisome, thermogenesis, retinol, pyruvate, and amino acids metabolism pathways are also affected by the mycotoxins.

The data also showed us that addition of antioxidants in feed was able to restore PRDX3, AGL, PYGL, fatty acids biosynthesis, endoplasmic reticulum state, peroxisome state, amino acid synthesis pathways, and, partially, OXPHOS mitochondrial subunits proteins expression. However, dosage studies need to be performed, excess of antioxidants might be also harmful, our data showing that CYP2C301, PPP4R4, COL18A1, UBASH3A and other proteins expression have been affected by antioxidants.

## 5. Materials and Methods

### 5.1. Animals, Treatment and Sampling

The experimental protocol was approved by the Ethics Committee (No. 118/2019) of the National Research-Development Institute for Animal Nutrition and Biology, Balotesti, Romania. Animals were cared for in accordance with the Romanian Law 43/2014 for handling and protection of animals used for experimental purposes and the EU Council Directive EC/63/2010 on the protection of farmed animals. The experiment was carried out within the experimental base of the National Research—Development Institute for Animal Biology and Nutrition, Balotesti, Romania.

In a 30-day feeding trial, a group of 40 weaned piglets of the TOPIGS-40 hybrid breed were divided into four groups. The piglets (females of 35 days) were randomly assigned, based on their body weight. Each group had 10 piglets, with two replicates of 5 pigs per pen. Feed for the control group (C) was produced in the pilot station of the National Research—Development Institute for Animal Biology and Nutrition, Balotesti, Romania consisting of 68.46% corn, 19% soya meal, 4% corn gluten, 5% milk replacer, 0.3% L-lysine, 0.1% DL-methionine, 1.57% limestone, 0.35% monocalcium phosphate, 0.1% salt, 0.1% choline premixes, and 1% vitamin-mineral premixes. The experimental groups were as follows: group A received the basal diet with a 5% addition of a 1:1 mixture of grape seed and sea buckthorn meal by-products, replacing corn and soya bean meal; group M received the basal diet with artificial contamination of aflatoxin B1 (AFB1) and ochratoxin A (OTA); and group AM received the basal diet with the added meal mixture and artificial contamination of AFB1 and OTA.

The mixture of mycotoxins was provided by the I.N.R.A Centre of Clermont-Ferrand, containing 30mg AFB1/kg and 230 mg OTA/kg, and was mixed into the experimental diets and screened by ELISA analysis. The final concentration for the experimental diets resulted from ELISA analysis, and was 62 µg/kg of AFB1 and 479 µg/kg OTA. The control diet and the experimental diets were also screened by ELISA for other mycotoxins (ZEA, DON, OTA, FBs) using ELISA Veratox kits according to the manufacturer’s instructions. Their concentrations were under the EU limits for pigs. The grape seed and sea buckthorn meal were provided by S.C. OLE-OMET S.R.L. and BIO-CATINA, Bucharest, Romania. The diet composition, fatty acid composition of grape seed and sea buckthorn, flavonoids and phenolic acids composition of byproducts, mineral composition of byproducts, animal performance, and biomarkers of liver and kidney function in plasma have been previously published by Popescu et al. [45].

Throughout the experiment, the piglets had ad libitum access to their assigned diet and water. At the end of the 30-day period, the piglets were fasted, electrically stunned, exsanguinated, and samples from liver were taken from four piglets per group, perfused with ice-cold saline solution to remove blood, and stored at −80 °C until isolation of the microsomal fraction. In accordance with ethical considerations and good scientific practice, efforts were taken to minimize the number of animals used in the study. Reducing the number of animals used in experiments enables researchers to collect data with the minimum number required [100].

### 5.2. Microsomal Fraction Isolation

A modified version of the method described by Rasmussen et al. [101] was used to isolate the microsomal fraction. In brief, 6 g of liver tissue was minced and homogenized in ice-cold Tris-sucrose buffer using a Glas-Col Tissue Homogenizing System. The crude homogenate obtained after centrifugation was further centrifuged at 100,000× *g* for 60 min at 4 °C to isolate the microsomal pellet. The purity of the microsomal and cytosolic fractions was assessed by immunoblotting. The details of the buffer used for suspending the microsomal pellet and the storage conditions are provided in the text. All the steps involved in the isolation process were performed on ice.

### 5.3. Trypsin Digestion

For proteomic analysis, the microsomes obtained were used. The protein concentration of the samples was calculated using the Bradford method [102], and a bovine serum albumin (BSA) standard curve was used for interpolation. To prepare the samples, a volume corresponding to 30 μg of protein was diluted in 50 mM NH_4_HCO_3_ up to 500 μL, resulting in a final concentration of 1.5 M urea. Subsequently, 75 ng of bovine serum albumin was added to the sample in 1.5 mL low binding tubes. The sample was then incubated with 25 μL of 100 mM DTT in 100 mM NH_4_HCO_3_ for 45 min at 37 °C. Alkylation was carried out by adding 26.25 μL of 300 mM IAA in 100 mM NH_4_HCO_3_ and incubated for 45 min at 37 °C in the dark. Trypsin digestion was performed using a trypsin solution of 1 μg/μL (Trypsin Gold, V528A, Promega, Madison, WI, USA), and 0.6 μL was added to the final volume (1:50) and left overnight at 37 °C with low shaking. The digestion was stopped by adding 10 μL of 10% trifluoroacetic acid. The peptides were then dried by speed-vacuum and resuspended in 30 μL of 2% acetonitrile with 0.1% formic acid. The sample was transferred to a clean autosampler vial with insert for LC-MS/MS analysis

### 5.4. LC-MS/MS Analysis

Peptides samples were analyzed by LC-MS/MS on AB SCIEX TRIPLE TOF 5600+ mass spectrometer and separated using NanoLC 425 system (Eksigent, Dublin, CA, USA) in a trap-elute configuration, including a trapping column C18, 5 μm, 300 μM ID, 25 mm long and analytical column Eksigent 5C18-CL-120, 300 μM ID, 150 mm length, connected to DuoSpray ion source (AB Sciex, Framingham, MA, USA). In the analysis, 5 μL of the peptides samples were loaded and cleaned on the trap column at 40 μL /min using Solvent A (0.1% formic acid) and eluted using a gradient from 5 to 80% Solvent B (0.1% formic acid in acetonitrile) over 105 min at 5 μL per min flow with column temperature of 55 °C. Each sample was run in triplicate. Ionization was achieved via electro spray ionization in positive ion mode with the ion spray voltage at 5500 V and source temperature at 200 °C. The TRIPLE TOF 5600+ was operated in DIA SWATH-MS mode, 64-variable-windows. The MS1 survey scan was acquired from 400–1250 *m*/*z*, MS2 spectra were acquired in high-sensitivity mode from 100–2000 *m*/*z*. The accumulation time was set to 0.25 s, and the ion scan was sampled at 45 ms time windows in high sensitivity mode, resulting in a final time of 5 s cycle. The mass spectrometry proteomics data have been deposited to the ProteomeXchange Consortium via the PRIDE [103] partner repository with the dataset identifier PXD040040.

### 5.5. Data Analysis

Protein identification from DIA data was performed label-free, library-free, using DIA-NN ver. 1.8.1 [47], with the raw spectra searched against the fasta file of the complete *Sus scrofa* proteome (UniProt, UP000008227, January 2023, 46 139) with a precursor m/z range 400–1250, and with trypsin as digestion enzyme. Data was searched with MBR enabled and robust LC as quantification strategy and FDR = 0.01. C carbamidomethylation and Ox (M) were selected as modifications. Retention time dependent normalization was used, quantitation was performed in DIA-NN with MaxLFQ algorithm.

Statistical and differential downstream analysis was performed using locally installed PolySTest version 1.3 (release) [104], which is available to download at: https://bitbucket.org/veitveit/polystest/src/master/, accessed on 10 January 2023, using as input unique gene matrix *tsv file resulted from DIA-NN. FDR adjusted *p*-value threshold was set to 0.01 and log_2_ fold change of −1 respective 1 to discriminate significantly down-respective up regulated proteins. We have selected in PolySTest to perform Limma (the most relevant for gene or protein expression data, as it performs well despite missing values in some replicates) and Miss test (which, according to the author, should improve the data overall significance by rescuing the significance of some low abundance proteins missing many values). However, it was not the case with our data; Limma was the only useful from PolyStest with our data. After statistical analysis, data, heatmap and expression profiles were generated.

Each identified gene from DIA-NN was converted and mapped onto its corresponding protein object. Proteins and gene products ID conversions were performed using R package ‘org.Ssc.eg.db’ in Bioconductor [105]. Downstream analysis and graphic plotting for proteomic data were primarily performed using the R studio platform (version 4.2.2).

For differential pathways expression analysis (PEA), the proteins list with Log_2_ fold change and statistical significance was exported from the PolyStest and imported in R for further analyzed in PathfindR version 1.6.4 [106]. Using a protein-protein interaction network (PIN) analysis approach, with PIN data for *Sus scrofa* from STRING (https://stringdb-static.org/download/protein.links.v11.5/, accessed on 10 January 2023), PathfindR outputs a table that represent enriched pathways identified from the protein list including a fold enrichment value of the pathway, the lowest and highest *p*-values generated from each iteration of the pathways analysis, and the upregulated and downregulated proteins from the input protein list for every pathway [106]. Also, we generated an enrichment chart and term graph for top 20 KEGG pathways and top 10 KEGG pathways, respectively, sorted by lowest *p* value.

KEGG pathway database (https://www.genome.jp/kegg/pathway.html, accessed on 10 January 2023), data integration and data visualization of the main biological process, Pathview R package version 1.38.0 [107] were used.

## Figures and Tables

**Table 1:** KEGG Pathways, antioxidants (A) vs. control (C) data sorted by lowest *p*.

ID	Term Description	FE	Lowest *p*	Highest *p*	Up_Regulated	Down_Regulated
ssc00071	Fatty acid degradation	11.4448	2.68 × 10^−18^	5.84 × 10^−16^	ALDH3A2	ACAT2, ACAA1, EHHADH, ACOX1, ACADM, ACADSB, ACADVL, ACSL1, ECI2, ALDH7A1
ssc00190	Oxidative phosphorylation	7.179012	1.45 × 10^−16^	1.45 × 10^−16^	ATP6V0D1	NDUFS1, NDUFS4, NDUFV1, NDUFA4, NDUFA6, NDUFA9, NDUFB7, NDUFB10, SDHA, SDHB, SDHC
ssc00280	Valine, leucine, and isoleucine degradation	9.076912	7.10 × 10^−15^	1.34 × 10^−14^	ALDH3A2	ACADM, ACADSB, EHHADH, ACAA1, PCCA, ALDH6A1, ALDH7A1, AUH, HMGCL, ACAT2
ssc03010	Ribosome	5.317787	6.60 × 10^−14^	1.25 × 10^−13^	RPS7, RPS19, RPL29	RPS3, RPS3A, RPS5, RPS13, RPL27A
ssc00020	Citrate cycle (TCA cycle)	9.97085	2.89 × 10^−13^	7.94 × 10^−13^	DLST	IDH2, IDH1, SUCLG1, SDHA, SDHB, SDHC, FH, PC, PCK2
ssc05208	Chemical carcinogenesis—reactive oxygen species	4.661696	3.90 × 10^−13^	3.90 × 10^−13^		NDUFV1, NDUFA4, NDUFA6, NDUFA9, NDUFB7, NDUFB10, NDUFS1, NDUFS4, SDHA, SDHB, SDHC, VDAC2, SLC25A4, SOD1, CAT
ssc04714	Thermogenesis	3.34506	3.45 × 10^−12^	3.45 × 10^−12^		ACSL1, NDUFS1, NDUFS4, NDUFV1, NDUFA4, NDUFA6, NDUFA9, NDUFB7, NDUFB10, SDHA, SDHB, SDHC, SLC25A20
ssc03040	Spliceosome	1.966853	1.33 × 10^−9^	1.33 × 10^−9^	SNRPA, HNRNPM, SRSF1, SRSF3, SRSF7	LSM8
ssc00650	Butanoate metabolism	8.973765	3.08 × 10^−9^	0.005032		ACAT2, EHHADH, ACSM4, ALDH5A1, HMGCL, BDH1
ssc00640	Propanoate metabolism	8.973765	6.16 × 10^−9^	6.16 × 10^−9^	ECHDC1	ACOX1, EHHADH, PCCA, SUCLG1, ALDH6A1
ssc04141	Protein processing in endoplasmic reticulum	3.625764	6.53 × 10^−9^	4.51 × 10^−8^	SEC63, RRBP1, HYOU1, DNAJB11, HSP90B1, PRKCSH, CANX, LMAN2, LMAN1, SEC13, SEC31A, PDIA4, ERP29, UBXN4, CAPN1	
ssc04146	Peroxisome	7.478138	1.54 × 10^−8^	2.42 × 10^−8^	PRDX1, XDH	ACOX1, HSD17B4, EHHADH, ACAA1, ACSL1, ECI2, CRAT, IDH2, IDH1, HMGCL, HAO1, CAT, SOD1
ssc00140	Steroid hormone biosynthesis	6.837155	9.82 × 10^−8^	9.82 × 10^−8^	HSD11B1, HSD17B7	AKR1D1, COMT
ssc04142	Lysosome	2.243441	2.56 × 10^−7^	2.56 × 10^−7^	ATP6V0D1, IGF2R, CLTC, AP1G1, AP1M1	SORT1
ssc00670	One carbon pool by folate	9.572016	2.88 × 10^−7^	4.52 × 10^−7^	GART	MTHFD1, AMT, ALDH1L1
ssc00330	Arginine and proline metabolism	5.438646	3.32 × 10^−6^	9.92 × 10^−6^	ALDH3A2	AGMAT, ALDH7A1, MAOB, ALDH4A1
ssc00250	Alanine, aspartate and glutamate metabolism	7.179012	6.58 × 10^−06^	6.58 × 10^−06^	GFPT1	ASS1, ASL, ALDH5A1, ALDH4A1, CPS1
ssc00380	Tryptophan metabolism	7.36309	6.78 × 10^−6^	1.22 × 10^−5^	DLST, ALDH3A2	KYNU, EHHADH, ACAT2, MAOB, ALDH7A1, CAT
ssc00620	Pyruvate metabolism	7.614104	1.56 × 10^−5^	1.56 × 10^−5^	ALDH3A2	ALDH7A1, GRHPR, PC, FH, PCK2, ACAT2
ssc00010	Glycolysis / Gluconeogenesis	4.102293	2.36 × 10^−5^	2.36 × 10^−5^	ALDH3A2	PGK1, ENO3, ALDH7A1, PGM2, PCK2
ssc00340	Histidine metabolism	5.98251	4.18 × 10^−5^	4.18 × 10^−5^	ALDH3A2	ALDH7A1, MAOB
ssc00410	beta-Alanine metabolism	7.038247	4.66 × 10^−5^	5.89 × 10^−5^	ALDH3A2	ALDH7A1, EHHADH, ACOX1, ALDH6A1
ssc00350	Tyrosine metabolism	5.630598	0.000129	0.000129	HPD	GSTZ1, COMT, MAOB
ssc00220	Arginine biosynthesis	7.97668	0.000249	0.000343	ACY1	ASS1, ASL, CPS1
ssc00230	Purine metabolism	1.734061	0.000383	0.000383	GART, ENTPD8, XDH	PGM2, AK3
ssc00310	Lysine degradation	5.776217	0.000454	0.000573	DLST, ALDH3A2	AASS, ALDH7A1, EHHADH, ACAT2, PLOD3
ssc00760	Nicotinate and nicotinamide metabolism	3.778428	0.000512	0.000512	NAMPT, NNMT	NNT
ssc00500	Starch and sucrose metabolism	2.518952	0.000512	0.000512	PYGL	PGM2
ssc04961	Endocrine and other factor-regulated calcium reabsorption	2.658893	0.000963	0.000963	AP2A1, AP2M1, CLTC	
ssc03320	PPAR signaling pathway	5.469724	0.000989	0.001231	APOC3	CYP27A1, ACSL1, EHHADH, ACAA1, ACOX1, ACADM, PCK2
ssc04918	Thyroid hormone synthesis	3.14869	0.001585	0.002762	HSP90B1, CANX, PDIA4	ASGR1, ASGR2
ssc00240	Pyrimidine metabolism	1.595336	0.002128	0.002128	ENTPD8	CMPK1
ssc00053	Ascorbate and aldarate metabolism	9.572016	0.002765	0.002765	ALDH3A2	ALDH7A1
ssc00630	Glyoxylate and dicarboxylate metabolism	7.97668	0.006161	0.007302		ACAT2, PCCA, HAO1, CAT, GRHPR, AMT
ssc00980	Metabolism of xenobiotics by cytochrome P450	2.658893	0.009935	0.009935	HSD11B1	
ssc00982	Drug metabolism—cytochrome P450	2.991255	0.023128	0.023128		MAOB
ssc00770	Pantothenate and CoA biosynthesis	1.99417	0.033874	0.033874	ALDH3A2	
ssc00830	Retinol metabolism	2.518952	0.046974	0.046974	RDH11	ALDH1A1

FE—Fold Enrichment; ID—KEGG ID; Term Description—pathway name from KEGG.

**Table 2:** KEGG Pathways, Mycotoxin (M) vs. Control (C) data sorted by lowest *p*.

ID	Term Description	FE	Lowest *p*	Highest *p*	Up_Regulated	Down_Regulated
ssc00640	Propanoate metabolism	19.85997	6.32 × 10^−14^	6.32 × 10^−14^	ACSS2, ECHDC1	BCKDHA, BCKDHB, DBT, EHHADH, PCCA, SUCLG1, SUCLG2, ALDH6A1
ssc00020	Citrate cycle (TCA cycle)	11.91598	8.70 × 10^−14^	4.11 × 10^−12^		ACO2, IDH2, SUCLG1, SUCLG2, SDHA, SDHB, SDHC, PC, PCK2
ssc00190	Oxidative phosphorylation	6.355191	3.61 × 10^−13^	5.32 × 10^−11^		NDUFS1, NDUFS4, NDUFV1, NDUFA4, NDUFA9, SDHA, SDHB, SDHC
ssc00100	Steroid biosynthesis	15.88798	4.51 × 10^−13^	4.51 × 10^−13^	SQLE, LSS, TM7SF2, DHCR24, DHCR7, LIPA	
ssc04141	Protein processing in endoplasmic reticulum	3.851631	1.66 × 10^−12^	1.66 × 10^−12^	HYOU1, DNAJC3, HSP90B1, GANAB, CANX, LMAN1, SEC13, SEC31A, BCAP31, RAD23B	RRBP1, VCP
ssc05208	Chemical carcinogenesis—reactive oxygen species	4.952097	2.01 × 10^−10^	1.37 × 10^−8^	AS3MT	NDUFV1, NDUFA4, NDUFA9, NDUFS1, NDUFS4, SDHA, SDHB, SDHC, SLC25A4, CAT, EGFR
ssc04714	Thermogenesis	3.758446	1.17 × 10^−9^	6.44 × 10^−8^	ACSL5, ACSL1	NDUFS1, NDUFS4, NDUFV1, NDUFA4, NDUFA9, SDHA, SDHB, SDHC, SLC25A20
ssc04146	Peroxisome	5.957992	6.36 × 10^−8^	6.36 × 10^−8^	ABCD3, ACSL5, ACSL1, PRDX1	EHHADH, ACAA1, IDH2, HAO1, CAT
ssc00071	Fatty acid degradation	9.670943	1.48 × 10^−7^	1.48 × 10^−7^	ACSL5, ACSL1, ALDH3A2	ACAA1, EHHADH, ADH4, ALDH7A1
ssc00280	Valine, leucine, and isoleucine degradation	13.14867	5.18 × 10^−7^	5.18 × 10^−7^	ALDH3A2, HMGCS1	BCKDHA, BCKDHB, DBT, EHHADH, ACAA1, PCCA, ALDH6A1, ALDH7A1, MCCC1, AUH
ssc00330	Arginine and proline metabolism	7.221808	7.16 × 10^−7^	0.000508	ALDH3A2	AGMAT, ALDH7A1, MAOB, ALDH4A1
ssc03320	PPAR signaling pathway	7.263076	1.41 × 10^−6^	1.41 × 10^−6^	HMGCS1, APOC3, ACSL5, ACSL1	CYP27A1, EHHADH, ACAA1, PCK2
ssc00650	Butanoate metabolism	11.91598	1.49 × 10^−6^	1.49 × 10^−6^	HMGCS1	EHHADH, ACSM3, ACSM4, ALDH5A1, BDH1
ssc00620	Pyruvate metabolism	8.66617	1.56 × 10^−5^	3.35 × 10^−5^	ACSS2, ALDH3A2	ADH4, ALDH7A1, PC, PCK2
ssc00360	Phenylalanine metabolism	7.943989	2.98 × 10^−5^	2.98 × 10^−5^	PAH	MAOB
ssc00500	Starch and sucrose metabolism	5.017256	3.68 × 10^−5^	3.68 × 10^−5^	GYS2, GBE1, PYGL	
ssc00340	Histidine metabolism	7.943989	4.18 × 10^−5^	7.30 × 10^−5^	ALDH3A2	ALDH7A1, MAOB
ssc00380	Tryptophan metabolism	6.110761	4.74 × 10^−5^	4.74 × 10^−5^	ALDH3A2	EHHADH, MAOB, ALDH7A1, CAT
ssc00830	Retinol metabolism	3.344838	9.19 × 10^−5^	9.19 × 10^−5^		ADH4, ALDH1A1
ssc00760	Nicotinate and nicotinamide metabolism	3.344838	0.00032	0.00032	NAMPT	NNT
ssc00010	Glycolysis / Gluconeogenesis	4.539422	0.001231	0.002146	ALDH3A2, ACSS2	ADH4, ALDH7A1, PCK2
ssc00410	beta-Alanine metabolism	7.476696	0.001402	0.001402	ALDH3A2	ALDH7A1, EHHADH, ALDH6A1
ssc00630	Glyoxylate and dicarboxylate metabolism	8.826655	0.00168	0.00168	ACSS2	ACO2, PCCA, HAO1, CAT
ssc04961	Endocrine and other factor-regulated calcium reabsorption	3.530662	0.002297	0.002297	AP2A1, AP2M1, CLTC	
ssc03010	Ribosome	1.765331	0.00234	0.00234	RPS7	RPS3
ssc00053	Ascorbate and aldarate metabolism	12.71038	0.002765	0.003869	ALDH3A2	ALDH7A1
ssc00061	Fatty acid biosynthesis	9.078845	0.003869	0.003869	ACSL5, ACSL1	
ssc00982	Drug metabolism—cytochrome P450	7.943989	0.007731	0.010815		ADH4, MAOB
ssc00230	Purine metabolism	0.921042	0.01688	0.01688	ENTPD8	AK3
ssc00350	Tyrosine metabolism	5.607522	0.024972	0.024972	FAH	MAOB, ADH4
ssc00220	Arginine biosynthesis	2.647996	0.025435	0.025435		CPS1
ssc04142	Lysosome	2.978996	0.031794	0.031794	LGMN, LIPA, CLTC, AP1G1, AP1M1	SORT1
ssc00592	alpha-Linolenic acid metabolism	3.971995	0.033874	0.033874		ACAA1
ssc00900	Terpenoid backbone biosynthesis	3.177596	0.045305	0.045305	HMGCS1	

FE—Fold Enrichment; ID—KEGG ID; Term Description—pathway name from KEGG.

**Table 3:** KEGG Pathways, antioxidants + mycotoxins (AM) vs. control (C) data sorted by lowest *p*.

ID	Term Description	FE	Lowest *p*	Highest *p*	Up_Regulated	Down_Regulated
ssc00071	Fatty acid degradation	12.96544	5.99 × 10^−14^	5.99 × 10^−14^	ACSL5, ALDH3A2	ACAA1, EHHADH, ACOX1, ACADM, ACADSB, CPT2, ECI2, ALDH7A1
ssc00190	Oxidative phosphorylation	6.709615	1.91 × 10^−13^	1.91 × 10^−13^	ATP6V0D1	NDUFS1, NDUFS4, NDUFV1, NDUFA4, NDUFA6, SDHA, SDHB, SDHC
ssc00280	Valine, leucine, and isoleucine degradation	11.31123	5.21 × 10^−13^	5.21 × 10^−13^	ALDH3A2	BCAT2, BCKDHA, DBT, ACADM, ACADSB, EHHADH, ACAA1, PCCA, ALDH7A1, MCCC1
ssc05208	Chemical carcinogenesis—reactive oxygen species	5.421911	1.07 × 10^−10^	1.07 × 10^−10^	AS3MT, CYP1A1, MAPK1	NDUFV1, NDUFA4, NDUFA6, NDUFS1, NDUFS4, SDHA, SDHB, SDHC, SLC25A4, SOD1, CYP1A2
ssc04714	Thermogenesis	3.527157	6.23 × 10^−10^	6.23 × 10^−10^	ACSL5	NDUFS1, NDUFS4, NDUFV1, NDUFA4, NDUFA6, SDHA, SDHB, SDHC, CPT2, SLC25A20
ssc03040	Spliceosome	2.451001	1.33 × 10^−9^	8.09 × 10^−8^	DDX39B, SNRNP70, HNRNPM, SRSF1, SRSF3, SRSF7	
ssc00980	Metabolism of xenobiotics by cytochrome P450	9.940171	4.13 × 10^−9^	4.13 × 10^−9^	CYP1A1, HSD11B1	CYP1A2
ssc00020	Citrate cycle (TCA cycle)	6.212607	5.01 × 10^−9^	5.01 × 10^−9^		SDHA, SDHB, SDHC, PC, PCK2
ssc04146	Peroxisome	6.212607	5.59 × 10^−9^	5.59 × 10^−9^	ACSL5, XDH	HACL1, ACOX1, EHHADH, ACAA1, PECR, ECI2, HAO1, SOD1
ssc00640	Propanoate metabolism	11.18269	6.16 × 10^−9^	6.16 × 10^−9^	ECHDC1	BCKDHA, DBT, ACOX1, EHHADH, PCCA
ssc04530	Tight junction	2.899217	6.31 × 10^−9^	6.31 × 10^−9^	HSPA4, EZR, MSN, ACTR3, ARPC1B, TUBA1A	MYL6
ssc00140	Steroid hormone biosynthesis	6.39011	3.28 × 10^−8^	3.28 × 10^−8^	HSD11B1, CYP1A1	CYP1A2
ssc00380	Tryptophan metabolism	8.0286	9.21 × 10^−8^	9.21 × 10^−8^	KYAT3, ALDH3A2, CYP1A1	EHHADH, MAOB, ALDH7A1, CYP1A2
ssc04610	Complement and coagulation cascades	2.485043	1.92 × 10^−6^	1.92 × 10^−6^	PROC, KNG1	SERPIND1
ssc00270	Cysteine and methionine metabolism	3.195055	2.00 × 10^−6^	2.00 × 10^−6^	KYAT3	BCAT2, TST
ssc04141	Protein processing in endoplasmic reticulum	3.614608	2.81 × 10^−6^	2.81 × 10^−6^	RRBP1, DNAJB11, CANX, PREB, SEC13, SEC31A, SEC23A, SEC24C, PDIA6, BCAP31, RAD23B	VCP
ssc00830	Retinol metabolism	4.708502	5.27 × 10^−6^	5.27 × 10^−6^	DHRS3, CYP1A1	CYP1A2
ssc04810	Regulation of actin cytoskeleton	2.274446	2.01 × 10^−5^	2.01 × 10^−5^	FN1, KNG1, MAPK1, PAK2, MYLK, ACTR3, ARPC1B, EZR, MSN	
ssc00340	Histidine metabolism	7.455128	2.09 × 10^−5^	2.09 × 10^−5^	ALDH3A2	ALDH7A1, MAOB
ssc00982	Drug metabolism—cytochrome P450	7.455128	6.37 × 10^−5^	6.37 × 10^−5^		MAOB, CYP1A2
ssc00330	Arginine and proline metabolism	5.421911	0.000146	0.000146	ALDH3A2	AGMAT, ALDH7A1, MAOB
ssc00620	Pyruvate metabolism	5.421911	0.000146	0.000146	ALDH3A2	ALDH7A1, PC, PCK2
ssc04142	Lysosome	3.727564	0.000504	0.000504	ATP6V0D1, PSAP, LAMP1, CLTC, AP1G1, AP1M1, AP1S1	SORT1
ssc03320	PPAR signaling pathway	7.668132	0.000593	0.000593	ACSL5	APOC3, CYP27A1, EHHADH, ACAA1, ACOX1, CPT2, ACADM, PCK2
ssc00010	Glycolysis/Gluconeogenesis	3.408059	0.000618	0.000618	ALDH3A2	PFKL, ALDH7A1, PCK2
ssc00053	Ascorbate and aldarate metabolism	11.92821	0.001844	0.001844	ALDH3A2	ALDH7A1
ssc00410	beta-Alanine metabolism	7.016591	0.003543	0.003543	ALDH3A2	ALDH7A1, EHHADH, ACOX1
ssc00591	Linoleic acid metabolism	4.260073	0.003869	0.003869		CYP1A2
ssc00230	Purine metabolism	1.296544	0.004906	0.004906	ENTPD8, XDH, AK1	
ssc03010	Ribosome	1.656695	0.005569	0.005569	RPS7, RPL29	
ssc04110	Cell cycle	0.828348	0.00558	0.00558	YWHAB, YWHAQ	
ssc04390	Hippo signaling pathway	0.764629	0.007107	0.007107	YWHAB, YWHAQ	
ssc04670	Leukocyte transendothelial migration	1.146943	0.007183	0.007183	EZR, MSN	
ssc00360	Phenylalanine metabolism	3.727564	0.007731	0.007731		MAOB
ssc00450	Selenocompound metabolism	3.727564	0.007731	0.007731	KYAT3	
ssc00100	Steroid biosynthesis	4.970085	0.01214	0.01214	TM7SF2, DHCR7	
ssc00220	Arginine biosynthesis	4.970085	0.018189	0.018189	ACY1	CPS1
ssc05205	Proteoglycans in cancer	1.114786	0.018403	0.018403	MAPK1, FN1, EZR, MSN	
ssc00310	Lysine degradation	5.141468	0.018658	0.018658	ALDH3A2	AASS, ALDH7A1, EHHADH, PLOD3
ssc00350	Tyrosine metabolism	1.754148	0.024972	0.024972		MAOB
ssc00630	Glyoxylate and dicarboxylate metabolism	3.31339	0.042049	0.042049		PCCA, HAO1

FE—Fold Enrichment; ID—KEGG ID; Term Description—pathway name from KEGG.

**Table 4:** Log_2_ fold change in the most significantly changed protein expression levels in the hepatic microsomal fraction of weaned piglets subjected to experimental diets. The control group (C) were fed with the basal diet. The experimental groups were fed as follows: the basal diet plus a mixture (1:1) of two byproducts (grapeseed and sea buckthorn meal) (A group), the basal diet artificially contaminated with AFB1 and OTA (M group), and the basal diet containing the mixture (1:1) of grapeseed and sea buckthorn meal and contaminated with the mix of AFB1 and OTA (AM group).

Protein Name	Log_2_FC A vs. C	Log_2_FC M vs. C	Log_2_FC AM vs. C	FDR A vs. C	FDR M vs. C	FDR AM vs. C
PRDX3 (peroxiredoxin 3)	1.258614154	1.529266	1.193005	2.55 × 10^−5^	6.32 × 10^−7^	7.74 × 10^−5^
AGL (amylo-alpha-1, 6-glucosidase, 4-alpha-glucanotransferase)	0.815832392	1.407128	1.036078	0.000266	8.55 × 10^−9^	9.87 × 10^−6^
PYGL (glycogen phosphorylase L)	0.486029891	1.451883	1.050166	0.036186	3.52 × 10^−7^	0.000158
CYP2C301 (cytochrome P450 family 2 subfamily C member 301)	0.85546147	0.416917	1.181411	0.034991	0.501544	0.002352
PPP4R4 (protein phosphatase 4 regulatory subunit 4)	1.609917423	0.978918	0.55693	0.001337	0.077077	0.346479
COL18A1 (collagen, type XVIII, alpha 1)	1.065421972	0.62971	0.11707	2.33 × 10^−10^	1.56 × 10^−5^	0.51763
UBASH3A (ubiquitin-associated and SH3 domain containing A)	1.079719585	0.262833	0.51158	0.000499	0.608022	0.160859
CNTRL (centriolin)	0.500587122	−0.07292	1.383758	0.271479	1	0.001199
MX1 (MX dynamin like GTPase 1)	−0.188707549	0.101622	1.40425	0.390073	0.011692	0.004935
PRCP (prolylcarboxypeptidase)	0.384300517	0.445297	1.038305	0.36654	0.502516	0.015257
RPL10 (ribosomal protein L10)	−0.115559617	0.484204	2.293986	0.962586	0.461103	0.017532
RTN3 (reticulon 3)	0.150853392	0.494111	1.48471	0.539651	0.021141	1.35 × 10^−6^
FGF22 (fibroblast growth factor 22)	0.340986241	1.202735	1.203546	0.449717	0.007073	0.003019
CFAP119 (cilia and flagella-associated protein 119)	−1.049586098	−0.36016	−0.02325	0.004013	0.428574	1
BDH1 (3-hydroxybutyrate dehydrogenase 1)	−1.137408133	−1.149	−0.8668	1.55 × 10^−10^	3.17 × 10^−11^	8.77 × 10^−8^
MPC2 (mitochondrial pyruvate carrier 2)	−0.589203615	−1.03423	−0.85404	0.001754	2.40 × 10^−7^	1.59 × 10^−5^
NDUFA6 (NADH:ubiquinone oxidoreductase subunit A6)	−0.822450977	−0.85757	−1.07614	0.034965	0.080535	0.014266
NDUFA8 (NADH:ubiquinone oxidoreductase subunit A8)	−0.814210357	−0.78726	−1.09126	0.018491	0.083708	0.007074
LOC100524873 (cytochrome b-c1 complex subunit 6, mt)	−1.052389525	−0.90247	−0.58203	0.011395	0.016436	0.072888
COX6A1 (cytochrome c oxidase subunit 6A1)	−1.115583202	−1.3148	−1.15173	0.003131	0.000488	0.01032
ATP5PB (ATP synthase peripheral stalk-membrane subunit b)	−0.831488452	−1.01989	−0.81775	9.84 × 10^−6^	1.52 × 10^−7^	1.80 × 10^−5^
ATP5PD (ATP synthase peripheral stalk subunit d)	−1.045104464	−1.24739	−0.76175	3.21 × 10^−8^	2.15 × 10^−10^	2.06 × 10^−5^
SDHC (succinate dehydrogenase complex subunit C)	−0.885137794	−1.01039	−0.95537	1.26 × 10^−7^	4.76 × 10^−9^	4.18 × 10^−8^
COX4I1 (cytochrome c oxidase subunit 4I1)	−0.822175296	−1.09644	−0.81609	2.02 × 10^−8^	7.46 × 10^−12^	3.34 × 10^−8^
ATP5MG (ATP synthase membrane subunit g)	−0.882912451	−1.03223	−0.8393	5.48 × 10^−8^	7.61 × 10^−10^	2.24 × 10^−7^
ATP5F1C (ATP synthase F1 subunit gamma)	−1.118273993	−1.23754	−1.06335	1.69 × 10^−8^	6.35 × 10^−10^	6.40 × 10^−8^
ATP5F1A (ATP synthase F1 subunit alpha)	−0.934445513	−1.11394	−0.90318	5.63 × 10^−8^	5.63 × 10^−10^	1.78 × 10^−7^
ATP5F1B (ATP synthase F1 subunit beta)	−0.957688895	−1.11768	−0.93965	1.57 × 10^−9^	1.94 × 10^−11^	5.81 × 10^−9^
ATP5F1D (ATP synthase F1 subunit delta)	−0.82234898	−1.05501	−0.77117	9.57 × 10^−7^	2.63 × 10^−9^	4.83 × 10^−6^
ATP5MF (ATP synthase membrane subunit f)	−0.824097973	−1.14139	−0.83169	0.000149	4.42 × 10^−7^	0.000163
ATP5ME (ATP synthase membrane subunit e)	−0.780345693	−1.09737	−0.82017	0.000598	3.42 × 10^−6^	0.000417
SIGLEC1 (sialic acid binding Ig like lectin 1)	−0.887453438	−1.0351	−0.66508	0.0002	2.13 × 10^−5^	0.006319
ATP5PO (ATP synthase peripheral stalk subunit OSCP)	−0.638347295	−1.01715	−0.46444	0.000147	1.64 × 10^−8^	0.00638
NDUFV3 (NADH:ubiquinone oxidoreductase subunit V3)	−0.763715269	−1.20934	−0.68555	0.002027	2.99 × 10^−6^	0.004935

**Table 5:** Log fold change for the mycotoxins effect (AM vs. A) of the most significantly changed protein expression levels in the hepatic microsomal fraction of weaned piglets subjected to experimental diets. The experimental groups were fed as follows: the basal diet plus a mixture (1:1) of two byproducts (grapeseed and sea buckthorn meal) (A group) and the basal diet containing the mixture (1:1) of grapeseed and sea buckthorn meal and contaminated with the mix of AFB1 and OTA (AM group).

Protein Name	Log_2_FC AM vs. A	FDR AM vs. A
RTN3 (reticulon 3)	1.333856488	9.52 × 10^−5^
HMGCS2 (3-hydroxy-3-methylglutaryl-CoA synthase 2)	1.444551447	0.000281205
RPS21 (ribosomal protein S21)	3.612517926	0.000827373
ERG28 (ergosterol biosynthesis 28 homolog)	1.975858933	0.000827702
PTPRC (receptor-type tyrosine-protein phosphatase C-like)	1.190814103	0.004299025
MX1 (MX dynamin like GTPase 1)	1.592957592	0.005482088
ANKRD17 (ankyrin repeat domain 17)	−1.300765515	0.00768479
CYP2C293 (cytochrome P450 family 2 subfamily C member 293)	1.629403286	0.008207595
VKORC1L1 (vitamin K epoxide reductase complex subunit 1 like 1)	1.966681037	0.008886381

**Table 6:** Log fold change for the antioxidants effect (AM vs. M) of the most significantly changed protein expression levels in the hepatic microsomal fraction of weaned piglets subjected to experimental diets. The experimental groups were fed as follows: the basal diet artificially contaminated with AFB1 and OTA (M group) and the basal diet containing the mixture (1:1) of grapeseed and sea buckthorn meal and contaminated with the mix of AFB1 and OTA (AM group).

Protein Name	Log_2_FC AM vs. M	FDR AM vs. M
RAB15 (RAB15, member RAS oncogene family)	4.664835366	2.37 × 10^−6^
HMGCS2 (3-hydroxy-3-methylglutaryl-CoA synthase 2)	1.509156307	0.000181408
LOC100519130 (N-fatty-acyl-amino acid synthase/hydrolase PM20D1)	1.210067496	0.000272316
RPS21 (ribosomal protein S21)	3.801722021	0.0002976
RIDA (reactive intermediate imine deaminase A homolog)	2.4589452	0.000315547
NDUFB11 (NADH:ubiquinone oxidoreductase subunit B11)	1.85720825	0.001889375
RTN3 (reticulon 3)	0.990599268	0.003772517
CNTRL (centriolin)	1.456675854	0.004866641
MX1 (MX dynamin like GTPase 1)	1.302627789	0.005956324
MRM1 (mitochondrial rRNA methyltransferase 1)	1.341183314	0.007101537
TMEM33 (transmembrane protein 33)	2.378798247	0.007127983
ERG28 (ergosterol biosynthesis 28 homolog)	1.480329632	0.00853595
VKORC1L1 (vitamin K epoxide reductase complex subunit 1 like 1)	1.934924004	0.00853595
C4BPA (complement component 4 binding protein, alpha)	1.426021648	0.00853595

## Data Availability

The data presented in this study are available on request from the corresponding author. The mass spectrometry proteomics data have been deposited to the ProteomeXchange Consortium via the PRIDE [103] partner repository with the dataset identifier PXD040040.

## Supplementary Materials

The following supporting information can be downloaded at: https://www.mdpi.com/article/10.3390/toxins15040299/s1. Table S1: Report from DIA-NN with unique genes matrix. Table S2: Results from PolySTest with log fold change threshold −0.5–0.5.
